# An Entropy-Balanced Orthogonal Learning Bamboo Forest Growth Optimization Algorithm with Quasi-Affine Transformation Evolutionary and Its Application in Capacitated Vehicle Routing Problem

**DOI:** 10.3390/e25111488

**Published:** 2023-10-27

**Authors:** Jeng-Shyang Pan, Xin-Yi Zhang, Shu-Chuan Chu, Ru-Yu Wang, Bor-Shyh Lin

**Affiliations:** 1 College of Computer Science and Engineering, Shandong University of Science and Technology, Qingdao 266590, China; jspan@cc.kuas.edu.tw (J.-S.P.); 201801061227@sdust.edu.cn (X.-Y.Z.); 202180060004@sdust.edu.cn (R.-Y.W.); 2 Department of Information Management, Chaoyang University of Technology, Taichung 41349, Taiwan; 3 Institute of Imaging and Biomedical Photonics, National Yang Ming Chiao Tung University, Tainan City 71150, Taiwan; borshyhlin@nycu.edu.tw

**Keywords:** heuristic optimization algorithm, bamboo forest growth optimization algorithm, orthogonal learning, quasi-affine transformation evolution, capacitated vehicle routing problem

## Abstract

The bamboo forest growth optimization (BFGO) algorithm combines the characteristics of the bamboo forest growth process with the optimization course of the algorithm. The algorithm performs well in dealing with optimization problems, but its exploitation ability is not outstanding. Therefore, a new heuristic algorithm named orthogonal learning quasi-affine transformation evolutionary bamboo forest growth optimization (OQBFGO) algorithm is proposed in this work. This algorithm combines the quasi-affine transformation evolution algorithm to expand the particle distribution range, a process of entropy increase that can significantly improve particle searchability. The algorithm also uses an orthogonal learning strategy to accurately aggregate particles from a chaotic state, which can be an entropy reduction process that can more accurately perform global development. OQBFGO algorithm, BFGO algorithm, quasi-affine transformation evolutionary bamboo growth optimization (QBFGO) algorithm, orthogonal learning bamboo growth optimization (OBFGO) algorithm, and three other mature algorithms are tested on the CEC2017 benchmark function. The experimental results show that the OQBFGO algorithm is superior to the above algorithms. Then, OQBFGO is used to solve the capacitated vehicle routing problem. The results show that OQBFGO can obtain better results than other algorithms.

## 1. Introduction

Aiming at the optimization algorithm, the heuristic algorithm [1] is proposed. In general, an optimization problem can be seen as a form of mathematical programming, searching for different combinations of variables in a specified range to obtain the ideal response to the issue, that is, when the running time is allowed to be long enough, to ensure an optimal solution [2,3]. The heuristic algorithm is different from the optimization algorithm [4]. It is an algorithm based on experience or intuitive construction. Within the acceptable cost range, it generally refers to the calculation time and space and gives a feasible solution to the problem. Generally, it is impossible to estimate the feasible solution. Heuristic algorithms can be classified into traditional heuristic algorithms and metaheuristic algorithms [5]. Traditional heuristic algorithms contain relaxation method, solution space reduction algorithm, stereotype method, local search algorithm [6] and so on. The metaheuristic algorithm is enhanced using the heuristic algorithm. Combining the random algorithm with local search, it can solve the best or satisfactory solution of complicated optimization problems. The metaheuristic algorithm is a mechanism based on computational intelligence to solve problems, so it is also called an intelligent optimization algorithm. Common metaheuristic algorithms include artificial neural network algorithm [7,8], simulated annealing algorithm [9], genetic algorithm [10,11], ant colony optimization algorithm [12], particle swarm optimization algorithm [13,14,15], artificial fish swarm algorithm [16,17], artificial bee colony algorithm [18,19,20], tabu search algorithm [21], differential evolution algorithm [22], etc. Jeng-Shyang Pan et al. proposed two novel algorithms based on State of Matter Search (SMS) algorithm [23]. Pei Hu et al. proposed a multi-surrogate assisted binary particle swarm optimization named as MS-assisted DBPSO [24]. Shu Chuan Chu et al. proposed a parallel fish migration optimization algorithm combining compact technology(PCFMO) [25]. Trong-The Nguyen et al. proposed an improved swarm algorithm method (ISA), which works well with picture segmentation, resulting in remarkable computing in terms of global convergence and resilience and preventing local optimization trapping [26]. Xingsi Xue et al. proposed a compact hybrid Evolutionary Algorithm (chEA) [27]. Huang Y et al. proposed a multiobjective particle swarm optimization (MOPSO) with diversity enhancing (DE) (MOPSO-DE) strategy [28]. Wang GG proposed a new kind of metaheuristic algorithm, called moth search (MS) algorithm [29]. Yu H. et al. proposed a surrogate-assisted hierarchical particle swarm optimizer [30]. In response to the proliferation of bio-inspired optimisation approaches in recent years, Molina et al. [31] propose to present two comprehensive, principle-based taxonomies and review and study more than three hundred papers dealing with naturally-inspired and bio-inspired algorithms, thus providing a critical summary of design trends and the similarities between them. Sörensen et al. [32] argue that most “novel” metaheuristics based on new metaphors are going backwards rather than forwards, and therefore call for a more rigorous evaluation of these approaches and point to some of the most promising avenues of research in the field of metaheuristics.

Metaheuristic algorithms are versatile optimization techniques, while entropy is a concept in information theory that gauges uncertainty and disorder in data. Despite their apparent disconnect, these two can synergize to tackle problems effectively. In certain optimization scenarios, particularly within metaheuristic algorithms, entropy can define “diversity” or “exploration”, preventing fixation on local optima. Here’s how these concepts intersect: 1. Diversity gauge. In metaheuristic algorithms, entropy quantifies solution set diversity. Higher entropy signals a wider range of solutions, indicating thorough exploration. Tracking entropy changes fine-tunes algorithm parameters, balancing exploration and exploitation. 2. Trade-off between exploration and exploitation. Metaheuristic algorithms juggle exploration and exploitation. Entropy gauges solution space uncertainty. Higher entropy implies more uncharted areas, pushing the algorithm to explore. Conversely, lower entropy suggests familiarity, guiding the algorithm using existing insights. 3. Multi-Objective optimization. Entropy measures solution distribution balance across objectives in multi-objective problems. Uniformly distributed solutions offer objective equilibrium, benefiting specific multi-objective optimization challenges.

The new metaheuristic algorithm for bamboo forest growth optimization algorithm [33] combines the growth characteristics of bamboo forest [34] with the optimization process of the algorithm. Bamboo is a tall tree-like grass plant, its growth rate is very fast, and its rapid growth process mainly occurs in its germination period [35]. For the first four years, the bamboo grows only 3 cm, however, from the fifth year onwards, the bamboo can grow at a rate of 30 cm per day, reaching 15 m in six weeks. In the soil, the root system of bamboo can stretch hundreds of square meters, and during the growth of bamboo shoots, bamboo can show rapid growth within a short time. Thus, the whole process of bamboo forest growth can be classified into the period when the bamboo whip expands underground and the upward growth period of bamboo shoots. Besides, the bamboo forest is made up of several bamboo whips, which are the underground stems of bamboo, usually relatively long and thin. The bamboo on a bamboo whip belongs to a group. Bamboo whip supplies its own energy by absorbing nutrients in the soil, thereby carrying out cell division and cell differentiation. The shoots growing on the bamboo whip will develop in two directions, one part will grow out of the ground and become new bamboo shoots, and the other part will grow horizontally and grow into new bamboo whips. When the metaheuristic algorithm is used to solve the problem, the two periods in the bamboo growth process, the period when the bamboo whip expands underground, and the period when bamboo shoots grow, can be used to correspond to the global exploration stage and the local development stage of the algorithm respectively. The BFGO algorithm is highly competitive in handling optimization problems, but its exploitation ability is not outstanding. Therefore we need to improve BFGO to enhance its exploitation ability.

The BFGO algorithm has some significant differences from the three algorithms, GA, PSO and ACO, in terms of the basic inspirations and the simulation objects, the iterative approach, and the parallelism. In terms of basic inspirations and simulated objects, BFGO draws inspiration from the growth process of bamboo. The subsurface expansion of bamboo whips and the growth of bamboo shoots correspond to global exploration and localized exploitation, respectively. GA emulates the process of natural evolution, using genetic encoding and genetic operators to search for the best solution. PSO mimics collective behavior observed in groups like birds or fish, where particles update their positions and velocities based on their own and the group’s information. ACO imitates the foraging behavior of ants. Ants choose paths based on pheromone information and heuristic knowledge. In the iterative approach, BFGO uses bamboo forest growth characteristics and differential equations of bamboo forest growth to adjust the positions of bamboo shoots on bamboo whips. GA generates new individuals in each generation through selection, crossover, and mutation operations. PSO operates by having each particle update its position and velocity based on the optimal position of itself and the population. ACO relies on ants selecting paths based on pheromone and heuristic information while releasing pheromone on the paths they traverse. Regarding parallelism, BFGO employs a parallel strategy, allowing individuals to communicate effectively with each other. GA can parallelize the processing of multiple individuals, with each individual undergoing crossover and mutation operations independently. PSO naturally exhibits parallelism, as each particle can be updated independently. ACO has a certain degree of parallelism, as multiple ants can explore different paths concurrently.

Quasi-Affine Transformation Evolution(QUATRE) algorithm using the quasi-affine transformation method is a group-based algorithm [36,37]. In terms of parameter optimization and large-scale optimization, this algorithm is superior to other algorithms. In addition, the algorithm has good cooperation, which can reduce the time complexity to a certain extent. Under the condition that the total number of times of entering the evaluation function remains unchanged, it can achieve better performance by increasing the overall size of particles to reduce the number of generations required for objective optimization. In general, the algorithm performs well in unimodal functions, multimodal functions, and high-dimensional optimization problems.

Experimental design is a mathematical theory and method, which is based on probability theory and mathematical statistics theory, economically and scientifically develops the experimental design, and effectively conducts statistical analysis on experimental data. The basic idea of orthogonal learning was proposed by Dr. Taguchi in Japan. Orthogonal learning strategy is widely used in production line design and process conditions, because it can output high-quality products while using few computing resources. Orthogonal arrays are an important tool in orthogonal learning strategy. Taguchi algorithm can use orthogonal arrays to improve its performance. In Taguchi’s algorithm, only two levels of orthogonal arrays are used to join the optimization process. An orthogonal array is first defined, since each column of the array will represent the value of a factor under consideration, and the factors in this orthogonal array can be manipulated independently.

Based on the bamboo forest growth optimization algorithm, this work proposes a new heuristic algorithm named Orthogonal Learning Bamboo Forest Growth Optimization Algorithm with quasi-affine transformation evolutionary (OQBFGO). This algorithm combines the quasi affine transformation evolution algorithm to expand the particle distribution range, which is a process of entropy increase and can greatly improve the particle search ability. The algorithm also uses an orthogonal learning strategy to accurately aggregate particles from a chaotic state, which is an entropy reduction process that can more accurately perform global development. Finally, a balance between exploration and development was achieved during the process of entropy increase and entropy decrease.

Finally, the improved algorithm is used to solve the capacitated vehicle routing problem (CVRP) [38,39], referred to as the vehicle routing problem. As the fundamental model of vehicle routing problem, this model usually only constrains the load and driving distance (or time) of vehicles, and there are almost no other constraints. For this problem, many algorithms have been applied to find the optimal solution of this problem [40,41]. Most of the other models’ various solving algorithms are also derived from this model [42]. At the end of this paper, the proposed new algorithm is used to solve this problem and has achieved good results. The main contributions are as follows.

1. For the first time, we combined the QUATRE algorithm with the BFGO algorithm. The new algorithm utilizes the evolutionary matrix from the Quasi-Affine Transformation Evolution algorithm to update particle positions, making particle movement more scientifically grounded, expanding the search space, and significantly improving the algorithm’s local search capabilities.

2. Innovatively, within the BFGO algorithm, we incorporated the use of an orthogonal learning strategy, enhancing the algorithm’s precision in global exploration, consequently improving its global development efficiency.

3. We tested the improved algorithm on both the CEC2013 and CEC2017 benchmark sets, comparing it with the original algorithm, various modifications of the original algorithm, and three other established algorithms, thus demonstrating the excellent performance of the new algorithm.

4. Building on the strong evidence of the enhanced algorithm’s effectiveness, we discretized the continuous OQBFGO algorithm and achieved success in solving the CVRP problem.

Other parts of the article are structured as follows. Section 2 will briefly introduce the theoretical basis of BFGO, QUATRE and Orthogonal Learning. Section 3 will introduce the specific process of the new algorithm OQBFGO in detail. Section 4 tests the algorithm on the CEC2017 benchmark function and shows the test results. Section 5 applies the new algorithm to the CVRP problem and compares its effect with the other five algorithms. In Section 6 of this paper, the work of this paper is summarized and the future direction is prospected.

## 2. Related Work

This part introduces the concept of bamboo forest optimization algorithm, quasi-affine transformation evolutionary algorithm, and orthogonal learning strategy in detail.

### 2.1. BFGO

The growth process of bamboo can be summarized as germination, shoot growth, rapid growth, adulthood, flowering and death stages [43]. In this part, the process of bamboo growth can be described by the optimization process of the algorithm. Taking bamboo root elongation, bamboo forest growth, and bamboo flowering as optimization principles, a new mathematical model was constructed. As a new metaheuristic algorithm, the bamboo forest optimization algorithm combines the characteristics reflected in the process of bamboo growth into the optimization procedure of the algorithm. The two periods in the bamboo growth process: the period when the bamboo whip expands underground and the period when bamboo shoots grow, can be used to correspond to the global exploration stage and the local development stage of the algorithm respectively.

In the extension stage of the bamboo whip, the nutrients in the soil beneficial to the growth of bamboo will be absorbed by the bamboo whip, and the energy will be stored, the meristematic tissue of the flagellar node of the bamboo whip will split, in the meanwhile, the underground buds of bamboo begin to extend randomly and expand the land it occupied. The buds on the bamboo whip have different development directions. Some of the more robust buds on the bamboo whip will try to grow out of the ground and grow into bamboo shoots, while the other part of the less robust buds will grow to the side, and these buds will eventually develop into new underground stems. The roots of bamboo growing under the ground also have different growth directions. As shown in Equations (1)–(5), the growth direction of roots includes the direction of group cognitive items, the direction of bamboo whip memory items and the direction of central items of bamboo forest.

(1) $$\begin{array}{c} X_{t+1}=\left\{\begin{array}{cc} X_{-}Gbest+m\times \left(q_{1}\times X_{-}Gbest-X_{t}\right)\times \cos\alpha , & r_{1}<0.4\\ X_{-}Pbest\left(k\right)+m\times \left(q_{1}\times X_{-}Pbest\left(k\right)-X_{t}\right)\times \cos\beta , & 0.4\leq r_{2}<0.7\\ X_{-}Cen\left(k\right)+m\times \left(q_{1}\times X_{-}Cen\left(k\right)-X_{t}\right)\times \cos\gamma , & \;\mathrm{else}\; \end{array}\right. \end{array}$$

(2) $$\begin{array}{c} \cos\alpha =\frac{X_{-}Gbest\;\times \;X_{t}}{\left|X_{t}\right|\;\times \;\left|X_{-}Gbest\right|} \end{array}$$

(3) $$\begin{array}{c} \cos\beta =\frac{X_{-}Pbest(k)\;\times \;X_{t}}{\left|X_{t}\right|\;\times \;\left|X_{-}Gbest\right|} \end{array}$$

(4) $$\begin{array}{c} \cos\gamma =\frac{X_{-}Cen(k)\;\times \;X_{t}}{\left|X_{t}\right|\;\times \;\left|X_{-}Gbest\right|} \end{array}$$

(5) $$\begin{array}{c} m=2-\frac{t}{T} \end{array}$$

where $X_{-}Gbest$ is used to represent the globally optimal individual among all particles, $X_{-}Pbest\left(k\right)$ is used to represent the best individual on the kth bamboo whip in the bamboo forest, and $X_{-}Cen\left(k\right)$ represents the center point of the kth bamboo whip in the bamboo forest. As shown in Equations (2)–(4), α, β and γ indicate the direction of the root system extending in different directions, which is the direction of the current individual on the group cognitive item, the bamboo whip memory item and the bamboo forest central item. In addition, q1 represents a random number with a value between 1 and 2. *m* is a number between 2 and 0 shown in Equation (5).

The bamboo shoot growth stage can be regarded as the selection stage of the bamboo forest growth optimization algorithm. In this stage, only a small part of the bamboo shoots can be unearthed and grow into bamboo, and the bamboo shoots that cannot be unearthed cannot grow into bamboo. Those bamboo shoots that have a chance to grow will get enough nutrients to grow rapidly in the short term. The differential equation of bamboo growth [44] is shown in Equation (6).

(6) $$\frac{dy}{dt}=\frac{a\times y}{t^{\lambda }\ln\left(\frac{c}{y}\right)}$$

Among them: λ>1, *a*, c>0 are parameters, *t* is the growth time of bamboo, and the height of bamboo is expressed by *y*. Its overall form is shown in Equation (7).

(7) $$y=c\times e^{-d}\times e^{\left(\frac{a}{\left(\lambda -1\right)\times t^{\left(\lambda -1\right)}}\right)}$$

Among them: *d* is the integral constant, which can be given according to the environment in which the tree grows. Then sort the equation as:

(8) $$X\left(\omega ,t\right)=SI\times e^{\frac{a}{k\times t^{k}}}$$

In Equation (8), SI is the maximum bamboo height under certain site conditions, which changes with site conditions; the two shape parameters of the bamboo growth model are expressed by *a* and *k*. Therefore, the value of SI can be given according to the growth environment of each bamboo.

According to Equations (6)–(8), we defined the temporary population of bamboo growth. The position is replaced only if the original group is not better than the individual. The updated equation is as follows,

(9) $$\begin{array}{c} X_{\mathrm{temp}\;}=\left\{\begin{array}{c} X_{t}+X_{-}Dist\times \Delta H\\ X_{t}-X_{-}Dist\times \Delta H \end{array}\right. \end{array}$$

(10) $$\begin{array}{c} X_{-}Dist=1-\left|\frac{1+X_{t}-X_{-}Cen\left(k\right)}{1+X_{-}Gbest-X_{-}Cen\left(k\right)}\right| \end{array}$$

(11) $$\Delta H=\frac{Q(t)-Q(t-1)}{X_{-}Gbest-X_{t}}$$

(12) $$Q\left(t\right)=X_{-}Gbest\times e^{\frac{a}{\varphi \times t^{\varphi }}}\times e^{-d}$$

In the above equations, $X_{-}Dist$ can be used to describe the proportional connection between the global optimum particle’s distance from the center particle and the distance from the current particle to the center particle. The difference between the two iterative growth is expressed in ΔH, the *t* generation’s cumulative growth is indicated in *Q*, and the value of *d* is between −1 and 1. The site conditions of bamboo can be expressed by *a* and φ.

### 2.2. QUATRE

The QUATRE algorithm, a novel evolutionary algorithm, enhances the cooperative ability of particles [45]. Its evolutionary formula, resembling the affine transformation in geometry, has led to its name as the evolutionary algorithm of quasi-affine transformation. The detailed evolution method is presented in Equation (13).

(13) $$\hat{X}\leftarrow B⨂M+\hat{X}⨂\bar{M}$$

In the above formula, the individual population matrix of particles is expressed by $\hat{X}$, $\hat{X}$ = ${\left[X_{1},X_{2},\ldots ,X_{ps}\right]}^{T}$, *i* ∈ [1,ps], ps stands for the size of the population, *B* represents a matrix that plays a guiding role in evolution, *M* stands for the co-evolutionary matrix, and ⨂ for the multiplication of the appropriate locations in the matrix.

As shown in Table 1, the evolution guidance matrix *B* has the following six generation methods. This paper chooses the first method.

*F* in the formula is a scale factor, which is essential for the change of particle position. In this work, we set *F* to 0.7. We randomly transform the row vector of *X* to obtain $X_{r1,G}$, $X_{r2,G}$, $X_{r3,G}$, $X_{r4,G}$ and $X_{r5,G}$. In this iteration, the best particle position is $X_{gbest,G}$.

*M* is the co-evolution matrix, which is transformed from the initial matrix through a series of changes. Matrix $M_{init}$ is a lower triangular matrix of *D* rows and *D* columns, and its element value is 1. Through two steps, we can convert $M_{init}$ to *M*. First, the operation is performed on the matrix $M_{init}$, which randomly rearranges the elements of all row vectors in the matrix $M_{init}$; in the second step, rearrange all row vectors of the matrix that has been changed through step 1. For example, when the population size ps is equal to the objective function dimension *D*, the above process is shown in Equation (14).

(14) $$M_{init}=\left[\begin{array}{cccc} 1 & & & \\ 1 & 1 & & \\ & & \ldots & \\ 1 & 1 & \ldots & 1 \end{array}\right]∼\left[\begin{array}{cccc} 1 & 1 & & \\ & \ldots & & \\ 1 & 1 & \ldots & 1\\ & & & 1 \end{array}\right]=M.$$

Generally speaking, the population individual size ps in the optimization algorithm is generally larger than the dimension *D* of the objective function. At this time, it is necessary to expand the number of rows of the matrix $M_{init}$ in Equation (14) from *D* rows to ps rows.

As shown in Equation (15), it shows the change when the population size ps is greater than the dimension *D*. At this time, the value of ps corresponds to the extended example when ps=s∗D+m and ps%D=m (% is the remainder operation).

(15) $$M_{init}=\left[\begin{array}{cccc} 1 & \\ 1 & 1 & \\ \; & \; & \cdots \\ 1 & 1 & \cdots & 1\\ 1 & \\ 1 & 1 & \\ \; & \; & \cdots & \\ 1 & 1 & \cdots & 1 \end{array}\right]∼\left[\begin{array}{cccc} 1 & 1 & \\ \; & \cdots & \\ 1 & 1 & \cdots & 1\\ \; & \; & 1 & \\ \; & \cdots & \\ 1 & \cdots & 1\\ \; & 1 & \\ 1 & \; & \cdots & 1 \end{array}\right]=M.$$

For example, when ps divided by *D* is equal to *s* and the remainder is *m*, the first s∗D rows of $M_{init}$ are stacked by this lower triangular matrix, stacked *s* times, and the last remaining *m* rows are the front of this lower triangular matrix *m* rows.

(16) $$M=\left[\begin{array}{cccc} 1 & \; & \\ 1 & 1 & \\ \; & \; & \cdots \\ 1 & 1 & \cdots & 1 \end{array}\right],\;\bar{M}=\left[\begin{array}{cccc} 0 & 1 & \cdots & 1\\ 0 & 0 & \cdots & 1\\ \; & \; & \cdots & 1\\ 0 & 0 & \cdots & 0 \end{array}\right].$$

Equation (16) shows the conversion process of the correlation matrix $\bar{M}$, which is to take the boolean value of the element in *M* inversely. For example, if the front is 1, the back will become 0, if the front is 0, the back will become 1.

### 2.3. Orthogonal Learning

In the process of scientific research, people often do many experiments to carry out a certain research. Experimental conditions generally include many factors. When the values of factors are different, the experimental results are also different. If every factor in the experiment process is taken through all its possible values, the total number of experiments to be done is the product of the number of values that can be taken by each factor. Therefore, this number may be large, exceeding the acceptable cost. For example, assuming that the result of an experiment is determined by the values of four factors *m*, *n*, *i*, and *j*, and each factor has 10 different values, then if we want to take each value into account, we need to do 10 × 10 × 10 × 10 = 10,000 experiments.

We can choose the most representative example of these values, so that we don’t have to try every case once, thus greatly reducing the number of tests required. In this process, we need to use orthogonal learning [46,47]. Through the orthogonal learning method, we can use reasonable experimental samples to determine the factors that constitute the best combination. OL methods are based on some orthogonal arrays for multifactorial and multilevel problems [48]. For example, suppose we design an orthogonal array table with seven dimensions in order to find the optimal combination of dimensions. As shown in Table 2, we can use $L_{8}\left(2^{7}\right)$ to represent an orthogonal table with 7 factors, 2 levels, and 8 combinations. In this formula, each factor corresponds to a column in the orthogonal table, and the range of values that each factor can take is expressed by level. OA has two main characteristics: 1. In each column of the OA table, each level of the same number of times will appear. 2. In any two columns of the orthogonal table, the number of combinations of the two levels will also appear the same time.

Which parameter values for trials are supposed to be taken into account are represented by the items in the orthogonal array displayed in Table 2. We can see that the elements in the orthogonal array in Table 2. have values of “1” or “2”, Where ‘1’ indicates the value of this factor is supposed to be taken from one of the candidate solutions, while ‘2’ means the value of this factor is supposed to be taken from another set of candidate solutions. For example, in the second combination in the table, we will obtain the values of factors $D_{1}$, $D_{2}$ and $D_{3}$ from the first set of solutions, and the values of factors $D_{4}$, $D_{5}$, $D_{6}$, and $D_{7}$ from the second set of solutions as a combination of experiments. Without using an orthogonal table, if we want to take every value into account, we need to conduct $2^{7}$ experiments, while using an orthogonal learning strategy, we will only need to conduct 8 experiments, which will greatly reduce the number of experiments.

## 3. The Proposed OQBFGO Algorithm

When combined with the growth characteristics of bamboo, the BFGO algorithm has better performance on complex problems, and it can balance the development capabilities during the exploration process. The OQBFGO algorithm proposed in this work adds an orthogonal learning strategy on the basis of the bamboo forest growth algorithm, which can carry out the global development more accurately and reduce the convergence time of the algorithm. Moreover, the algorithm also adds the QUATRE algorithm, which is helpful, and the search range is greatly expanded. When improving metaheuristic algorithms, it’s essential to choose strategies based on the optimization characteristics of each stage. In the early stages of the algorithm, the entire population should quickly explore a wide decision space in a distributed manner. In the mid-stage, perturbations and fine-tuning should be conducted near potential optimal positions to develop more likely extremal points. In the later stages, a balance between exploration and exploitation is crucial, avoiding both excessive dispersion and over-concentration. The appropriate combination of exploration and exploitation strategies is essential for achieving the best results. Algorithm 1 shows the pseudocode of OQBFGO.


**Algorithm 1** Pseudocode of OQBFGO  Initialize population size *N*, dimension *D*, and number of bamboo whips *K*.  Initialize the bamboo position $X_{t}$ and divide the population into *K* groups according to the fitness.  Update global optimal $X_{Gbest}$ and intra group optimal $X_{p}$.  **while** t < T **do**  **if** t > 10 and $X_{Gbest}$ not updat **then**   select some individuals from the elite library to update, and update $X_{p}$, $X_{Gbest}$.  **end if**  **if** $X_{p}$ not update **then**   reshuffle the individuals and then group them.  **end if**  Update $X_{t}$ according to Equations (1)–(5), sort and update $X_{p}$ and $X_{Gbest}$.  Update $X_{temp}$ according to Equations (6)–(12) and update $X_{t}$, $X_{p}$ and $X_{Gbest}$.  Carry out an orthogonal learning on the average value of $X_{p}$ obtained at this time and $X_{Gbest}$.  Update $X_{p}$, $X_{Gbest}$ and $X_{t}$.  Update the current coordinates of the particle according to Equations (13)–(16).  **end while**


Step 1: Initialization. Same as the original BFGO, the particle position is first initialized to generate a population position of size N∗D. The specific form is shown in Equation (17), where the number of particles is represented by *N* and the number of dimensions by *D*.

(17) $$X={\left[x_{1},x_{2},\ldots ,x_{ps}\right]}^{T}$$

Step 2: Find the fitness values of all particles, and divide all particles equally into *K* groups, that is, *K* bamboo whips and each bamboo whip has *n* bamboo buds. Traverse *K* bamboo whips, update the optimal $X_{p}$ in the group as needed, and then update the global optimal $X_{Gbest}$.

Step 3: Enter the iteration, first judge whether the algorithm has iterated enough times and the global optimal position $X_{Gbest}$ has not been updated, if yes, select some individuals from the elite library to update, and update $X_{p}$, $X_{Gbest}$.

Step 4: Determine whether the optimal $X_{p}$ within the group of all groups has not been updated, if so, reshuffle the individuals and then group them.

Step 5: Bamboo Whip Extension Stage: Update $X_{t}$ according to the Equations (1)–(5) mentioned above, and sort and update $X_{p}$ and $X_{Gbest}$.

Step 6: Bamboo forest growth stage. Update the position of the bamboo according to Equations (6)–(10) mentioned above, and then update the optimal $X_{p}$ within the group and the global optimal $X_{Gbest}$.

Step 7: An orthogonal learning is carried out between the global optimal position $X_{Gbest}$ obtained after the above steps and the average value of the optimal position $X_{p}$ of each group, and $X_{p}$, $X_{Gbest}$ and $X_{t}$ are updated.

Step 8: Update the current coordinates of the particle according to Equations (13)–(16) and use it as the initial position of the next iteration.

## 4. Simulation Experiment and Result Analysis

Select 29 functions of CEC2017 [49] as test functions to test the optimization effect of OQBFGO. The first and third functions are unimodal functions, the fourth to tenth functions are simple multimodal functions, the eleventh to twentieth functions are mixed functions, and twenty-first to thirtieth functions are combined functions.

### 4.1. Comparison with the Original Algorithm and Other Improvements of the Original Algorithm

Next, we tested the improved algorithm OQBFGO and bamboo forest growth optimization (BFGO), quasi-affine transformation evolutionary bamboo forest growth optimization (QBFGO), and orthogonal learning bamboo forest growth optimization (OBFGO) on CEC2017. To ensure fairness, each algorithm entered the function 10,000 times. Test dimensions include 10D, 30D, and 50D.

The test results for the new method suggested in this research, the original algorithm, and the improved algorithm of the original algorithm on CEC2017 test function are displayed in Table 3, Table 4 and Table 5, where mean represents the mean value of fitness, std represents the standard deviation, and the best results are shown in bold to make the results more intuitive. The number of times each algorithm beats the others is expressed in win. In Table 3, Table 4 and Table 5, the first column represents the original BFGO algorithm, while the second, third, and fourth columns depict the ablative experiments we conducted to improve this algorithm. The purpose of these experiments was to demonstrate the effectiveness of each strategy we introduced, all of which contributed to the superior performance of the OQBFGO algorithm. From the table, it is evident that the optimization results in the second, third, and fourth columns are significantly better than those in the first column. Furthermore, the fourth column represents an improvement over the second and third columns. The minor differences in the latter three columns are primarily due to the substantial improvements made by adding one strategy, and the subsequent strategies were aimed at achieving even more efficient optimization results based on this already excellent performance.

As shown in the table, the new algorithm proposed in this paper is better than the original algorithm and other improvements of the original algorithm, the new algorithm improved by using the orthogonal learning strategy and affine transformation algorithm has won 13 times in 10 dimensions, 14 times in 30 dimensions and 15 times in 50 dimensions. Therefore, at high latitudes, the new algorithm works better.

### 4.2. Comparison with Mature Algorithms

In addition, we also compared the performance of the improved algorithm OQBFGO with three other mature algorithms such as PSO, GWO [50,51], and DE [52,53] on CEC2017. Similarly, to ensure fairness, each algorithm enters the function 10,000 times. Test dimensions include 10D, 30D, and 50D. You can see the results in Table 6, Table 7 and Table 8. In those tables, mean represents the mean value of fitness, std represents the standard deviation, and the best results are shown in bold to make the results more intuitive. The number of times each algorithm beats the others is expressed in win.

As you can see from the tables above, the new algorithm OQBFGO has won 25 times on 10 dimensions, 18 times on 30 dimensions, and 18 times on 50 dimensions. The new algorithm works better than the other three mature algorithms.

### 4.3. Experiments on the CEC2013 Test Set

In order to further prove the effectiveness of the algorithm, this paper also conducts experiments on the CEC2013 test set, and the results are shown in Table 9, Table 10, Table 11 and Table 12, where Table 9 and Table 10 show the results of comparing the improved algorithm with the original algorithm and other improvements of the original algorithm, with dimensions of 30 and 50 dimensions, respectively, and Table 11 and Table 12 represent the results of comparing the improved algorithm with the other three mature algorithms, with dimensions of 30 and 50 dimensions, respectively. In the first two tables, OQBFGO wins 13 times in 30 dimensions and 17 times in 50 dimensions, and in the last two tables, OQBFGO wins 17 times in 30 dimensions and 16 times in 50 dimensions.

## 5. Application of OQBFGO in CVRP

### 5.1. Details of CVRP

There is a group of vehicles to serve several customers. The number of customers is *N*, and the quantity of goods required by each customer is different. The number of vehicles is *K*, and the distribution center is *D*. The *K* cars start from *D* and serve each customer and only once. All cars start at *D* and end up at *D*. The problem is to drive the car a minimum distance.

(18) $$\begin{array}{c} \min f\left(c_{ij}\right)=\sum_{i=0}^{N}\sum_{j=0}^{N}\sum_{k=0}^{K}\left(c_{ij}x_{ijk}\right)\\ \;\mathrm{st}\;\\ \sum_{k=1}^{K}y_{ki}=\left\{\begin{array}{c} 1,(i=1,2,\ldots ,N)\\ m(m\leq K),i=0 \end{array}\right.\\ \sum_{i=1}^{N}x_{irk}-\sum_{j=1}^{N}x_{rjk}=0,r=1,\ldots ,N,k=1,2,\ldots ,m\\ \sum_{i=1}^{N}x_{i0k}=\sum_{j=1}^{N}x_{0jk}=1,k=1,2,\ldots ,m\\ \sum_{i=1}^{N}\;g_{i}y_{ki}\leq q,k=1,2,\ldots ,m\\ \sum_{i=0}^{N}x_{ijk}=y_{kj},i\neq j,j=0,1,2,\ldots ,N\\ x_{ijk}=\left\{\begin{array}{c} 1,\;\mathrm{Vehicle}\;k\;\mathrm{is}\;\mathrm{transported}\;\mathrm{from}\;i\;\mathrm{to}\;j\\ 0,\;\mathrm{else}\; \end{array}\right.\\ y_{ki}=\left\{\begin{array}{c} 1,\;\mathrm{Customer}\;i\;\mathrm{is}\;\mathrm{served}\;\mathrm{by}\;\mathrm{vehicle}\;k\\ 0,\;\mathrm{else}\; \end{array}\right. \end{array}$$

In Equation (18), whether the delivery vehicle passes through the line between the two customers *j* to *i* is represented by $x_{ijk}$, 0 means no pass, and 1 means pass; whether the vehicle *k* will carry the goods for customer *i*, this is denoted by $y_{ki}$, 0 is no, 1 is yes; the number of vehicles used is expressed in *m*; the cost required for the vehicle to transport goods from customer *i* to customer *j* is expressed in $c_{ij}$; the quantity of goods required by customer *i* is expressed in $g_{i}$; the quantity that the vehicle can carry is expressed in *q*; the number of customers is represented by *N*, where the number of distribution centers is 0; the number of cars at distribution centers is denoted by *K*.

### 5.2. Simulation Experiment and Result Analysis

The CVRP calculation example used in this paper comes from the data set of Augerat et al., data structure is described below. Any data file, such as A-n32-k5, is divided into the following parts:

(1) Data information, including the following information. The data set’s name is A-n32-k5; the minimum number of cars is 5, and the optimal route length is 784; the problem type is CVRP; the number of customers, that is, the dimension is 32; the maximum vehicle load is 100 units.

(2) Node coordinates, this part gives the node number and X, Y coordinates. Based on this information, the two-dimensional Euclidean distance between two nodes can be calculated.

(3) Node demand, this part gives the demand of each node.

Next, we tested BFGO, QUATRE, PSO, GWO, DE, and OQBFGO on these ten calculation examples. To ensure fairness, the number of times the six algorithms entered the function was 20,000 times. As can be seen from Table 13, when OQBFGO is used to solve CVRP, better results can be achieved than the other five algorithms.

**Figure 1:**
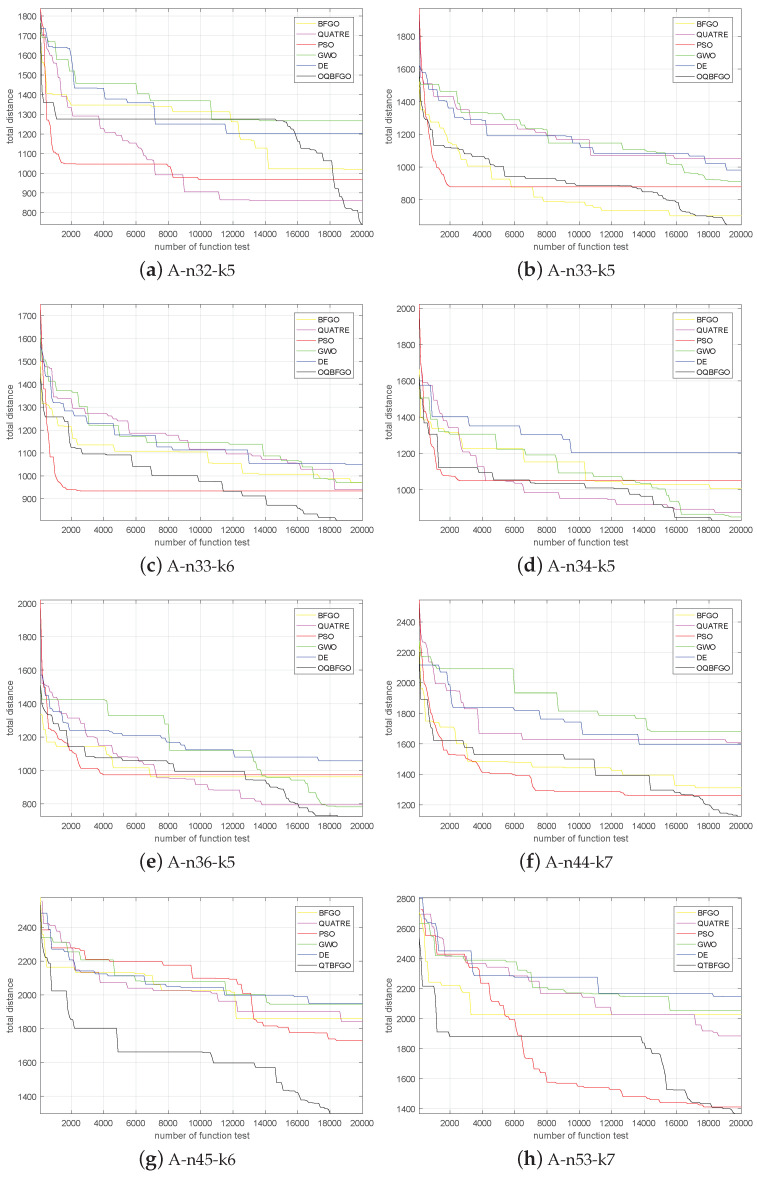
Convergence curve for solving CVRP problem.

The convergence curve is shown in Figure 1.

As shown in the figure, the OQBFGO algorithm can achieve the best results on ten calculation examples.

## 6. Conclusions

The OQBFGO algorithm proposed in this work adds an orthogonal learning strategy on the basis of the bamboo forest growth algorithm, which can perform global development more accurately. In addition, in order to improve the searching ability of particles, the algorithm also adds a quasi-affine transformation evolution algorithm. Next, we compare OQBFGO algorithm with the original algorithm BFGO and other improvements of the original algorithm: BFGO combined with QUATRE algorithm, BFGO combined with Taguchi strategy, in addition, also compared with three mature algorithms such as PSO, GWO, and DE, the test results on 29 functions of CEC2017 show that compared with the other six algorithms, OQBFGO can achieve better results.

Finally, we apply the OQBFGO algorithm to CVRP. Through the test on 10 groups of CVRP calculation examples, OQBFGO is compared with BFGO, QUATRE, PSO, GWO, and DE. It can be seen from the experimental results that OQBFGO performs better in solving CVRP, that is, OQBFGO can always search for the shortest effective path. However, OQBFGO still has problems such as long running time and slow convergence speed. These problems are also improvement directions that can be considered in the future.

## Figures and Tables

**Table 1:** The six schemes of matrix B calculation in QUATRE algorithm.

No.	QUATRE Variants	Equation
1	QUATRE/best/1	B = $X_{gbest,G}+F*\left(X_{r1,G}-X_{r2,G}\right)$
2	QUATRE/best/2	B = $X_{gbest,G}$ + $F*\left(X_{r1,G}-X_{r2,G}\right)+F*\left(X_{r3,G}-X_{r4,G}\right)$
3	QUATRE/rand/1	B = $X_{r1,G}+F*\left(X_{r2,G}-X_{r3,G}\right)$
4	QUATRE/rand/2	B = $X_{r1,G}+F*\left(X_{r2,G}-X_{r3,G}\right)+F*\left(X_{r4,G}-X_{r5,G}\right)$
5	QUATRE/target/1	B = $X_{G}+F*\left(X_{r1,G}-X_{r2,G}\right)$
6	QUATRE/target/2	B = $X_{G}+F*\left(X_{r1,G}-X_{r2,G}\right)+F*\left(X_{r3,G}-X_{r4,G}\right)$

**Table 2:** Orthogonal array with 7 factors, 8 combinations and 2 levels.

Combination Number	Dimensional Factors						
	$D_{1}$	$D_{2}$	$D_{3}$	$D_{4}$	$D_{5}$	$D_{6}$	$D_{7}$
1	1	1	1	1	1	1	1
2	1	1	1	2	2	2	2
3	1	2	2	1	1	2	2
4	1	2	2	2	2	1	1
5	2	1	2	1	2	1	2
6	2	1	2	2	1	2	1
7	2	2	1	1	2	2	1
8	2	2	1	2	1	1	2

**Table 3:** Use the test results of CEC2017 on 10 dimensions and compare with the original algorithm and other improvements of the original algorithm (bold font indicates optimal data).

Function	BFGO		QBFGO		OBFGO		OQBFGO	
	Mean	Std	Mean	Std	Mean	Std	Mean	Std
F1	2.30 × $10^{04}$	2.67 × $10^{04}$	2.08 × $10^{04}$	1.75 × $10^{04}$	**9.19 × $10^{03}$**	**9.10 × $10^{03}$**	9.76 × $10^{03}$	1.28 × $10^{04}$
F3	3.00 × $10^{02}$	4.98 × $10^{-01}$	**3.00 × $10^{02}$**	**1.52 × $10^{-01}$**	3.00 × $10^{02}$	1.54 × $10^{-01}$	3.00 × $10^{02}$	5.65 × $10^{-01}$
F4	4.08 × $10^{02}$	1.27 × $10^{01}$	**4.07 × $10^{02}$**	**2.34 × $10^{00}$**	4.11 × $10^{02}$	1.64 × $10^{01}$	4.10 × $10^{02}$	1.72 × $10^{01}$
F5	5.17 × $10^{02}$	6.54 × $10^{00}$	**5.14 × $10^{02}$**	**6.72 × $10^{00}$**	5.20 × $10^{02}$	7.77 × $10^{00}$	5.15 × $10^{02}$	5.23 × $10^{00}$
F6	6.03 × $10^{02}$	2.03 × $10^{00}$	6.01 × $10^{02}$	9.90 × $10^{-01}$	6.04 × $10^{02}$	4.00 × $10^{00}$	**6.01 × $10^{02}$**	**1.01 × $10^{00}$**
F7	7.31 × $10^{02}$	1.42 × $10^{01}$	**7.26 × $10^{02}$**	**9.67 × $10^{00}$**	7.29 × $10^{02}$	1.19 × $10^{01}$	7.27 × $10^{02}$	7.67 × $10^{00}$
F8	8.18 × $10^{02}$	7.74 × $10^{00}$	8.16 × $10^{02}$	5.89 × $10^{00}$	8.15 × $10^{02}$	6.66 × $10^{00}$	**8.16 × $10^{02}$**	**5.45 × $10^{00}$**
F9	9.05 × $10^{02}$	9.17 × $10^{00}$	9.03 × $10^{02}$	4.12 × $10^{00}$	9.06 × $10^{02}$	9.37 × $10^{00}$	**9.01 × $10^{02}$**	**1.23 × $10^{00}$**
F10	1.73 × $10^{03}$	2.67 × $10^{02}$	**1.70 × $10^{03}$**	**2.16 × $10^{02}$**	1.80 × $10^{03}$	2.48 × $10^{02}$	1.68 × $10^{03}$	3.54 × $10^{02}$
F11	1.14 × $10^{03}$	2.67 × $10^{01}$	1.13 × $10^{03}$	1.36 × $10^{01}$	1.14 × $10^{03}$	2.66 × $10^{01}$	**1.13 × $10^{03}$**	**1.53 × $10^{01}$**
F12	2.75 × $10^{04}$	2.08 × $10^{04}$	3.54 × $10^{04}$	5.59 × $10^{04}$	4.19 × $10^{04}$	1.00 × $10^{05}$	**1.41 × $10^{04}$**	**1.17 × $10^{04}$**
F13	5.80 × $10^{03}$	4.78 × $10^{03}$	6.21 × $10^{03}$	6.25 × $10^{03}$	7.00 × $10^{03}$	7.57 × $10^{03}$	**4.04 × $10^{03}$**	**3.80 × $10^{03}$**
F14	1.47 × $10^{03}$	1.37 × $10^{02}$	1.43 × $10^{03}$	1.12 × $10^{01}$	1.45 × $10^{03}$	1.75 × $10^{01}$	**1.43 × $10^{03}$**	**9.94 × $10^{00}$**
F15	1.60 × $10^{03}$	9.96 × $10^{01}$	**1.52 × $10^{03}$**	**8.41 × $10^{00}$**	1.59 × $10^{03}$	8.00 × $10^{01}$	1.53 × $10^{03}$	1.88 × $10^{01}$
F16	1.76 × $10^{03}$	1.22 × $10^{02}$	1.68 × $10^{03}$	8.28 × $10^{01}$	1.75 × $10^{03}$	8.32 × $10^{01}$	**1.67 × $10^{03}$**	**6.66 × $10^{01}$**
F17	1.75 × $10^{03}$	2.13 × $10^{01}$	1.74 × $10^{03}$	2.05 × $10^{01}$	1.75 × $10^{03}$	2.71 × $10^{01}$	**1.74 × $10^{03}$**	**1.80 × $10^{01}$**
F18	1.13 × $10^{04}$	8.68 × $10^{03}$	**8.22 × $10^{03}$**	**7.44 × $10^{03}$**	1.28 × $10^{04}$	1.39 × $10^{04}$	9.67 × $10^{03}$	8.38 × $10^{03}$
F19	2.42 × $10^{03}$	1.51 × $10^{03}$	1.91 × $10^{03}$	1.08 × $10^{01}$	2.63 × $10^{03}$	2.09 × $10^{03}$	**1.91 × $10^{03}$**	**8.89 × $10^{00}$**
F20	**2.04 × $10^{03}$**	**1.49 × $10^{01}$**	2.03 × $10^{03}$	1.61 × $10^{01}$	2.05 × $10^{03}$	2.47 × $10^{01}$	2.04 × $10^{03}$	3.34 × $10^{01}$
F21	2.24 × $10^{03}$	5.50 × $10^{01}$	**2.20 × $10^{03}$**	**1.22 × $10^{00}$**	2.24 × $10^{03}$	5.27 × $10^{01}$	2.21 × $10^{03}$	3.56 × $10^{01}$
F22	2.30 × $10^{03}$	1.48 × $10^{01}$	2.30 × $10^{03}$	1.64 × $10^{01}$	**2.30 × $10^{03}$**	**1.45 × $10^{01}$**	2.30 × $10^{03}$	2.52 × $10^{01}$
F23	2.62 × $10^{03}$	7.62 × $10^{00}$	**2.61 × $10^{03}$**	**6.27 × $10^{00}$**	2.62 × $10^{03}$	7.98 × $10^{00}$	2.62 × $10^{03}$	7.99 × $10^{00}$
F24	**2.71 × $10^{03}$**	**9.48 × $10^{01}$**	2.58 × $10^{03}$	1.18 × $10^{02}$	2.73 × $10^{03}$	7.82 × $10^{01}$	2.63 × $10^{03}$	1.27 × $10^{02}$
F25	2.93 × $10^{03}$	2.27 × $10^{01}$	2.93 × $10^{03}$	2.37 × $10^{01}$	2.93 × $10^{03}$	2.30 × $10^{01}$	**2.93 × $10^{03}$**	**2.25 × $10^{01}$**
F26	2.98 × $10^{03}$	9.39 × $10^{01}$	2.91 × $10^{03}$	7.89 × $10^{01}$	2.95 × $10^{03}$	1.05 × $10^{02}$	**2.95 × $10^{03}$**	**7.80 × $10^{01}$**
F27	3.08 × $10^{03}$	3.17 × $10^{01}$	**3.08 × $10^{03}$**	**1.14 × $10^{01}$**	3.08 × $10^{03}$	3.14 × $10^{01}$	3.08 × $10^{03}$	2.30 × $10^{01}$
F28	3.27 × $10^{03}$	5.02 × $10^{00}$	**3.27 × $10^{03}$**	**1.07 × $10^{-03}$**	3.27 × $10^{03}$	2.01 × $10^{01}$	3.26 × $10^{03}$	4.03 × $10^{01}$
F29	3.20 × $10^{03}$	4.65 × $10^{01}$	3.18 × $10^{03}$	3.39 × $10^{01}$	3.20 × $10^{03}$	4.53 × $10^{01}$	**3.18 × $10^{03}$**	**2.63 × $10^{01}$**
F30	4.54 × $10^{03}$	3.81 × $10^{03}$	**3.37 × $10^{03}$**	**1.89 × $10^{02}$**	4.29 × $10^{03}$	3.05 × $10^{03}$	3.41 × $10^{03}$	2.62 × $10^{02}$
**Win**	**2**		**12**		**2**		**13**	

**Table 4:** Use the test results of CEC2017 on 30 dimensions and compare with the original algorithm and other improvements of the original algorithm (bold font indicates optimal data).

Function	BFGO		QBFGO		OBFGO		OQBFGO	
	Mean	Std	Mean	Std	Mean	Std	Mean	Std
F1	8.61 × $10^{06}$	1.40 × $10^{07}$	4.45 × $10^{07}$	6.68 × $10^{07}$	9.09 × $10^{05}$	1.20 × $10^{06}$	**7.97 × $10^{06}$**	**9.72 × $10^{06}$**
F3	8.08 × $10^{03}$	3.99 × $10^{03}$	6.26 × $10^{03}$	2.13 × $10^{03}$	4.85 × $10^{03}$	1.96 × $10^{03}$	**4.22 × $10^{03}$**	**1.78 × $10^{03}$**
F4	5.04 × $10^{02}$	3.52 × $10^{01}$	5.17 × $10^{02}$	3.42 × $10^{01}$	**4.79 × $10^{02}$**	**3.23 × $10^{01}$**	4.97 × $10^{02}$	2.30 × $10^{01}$
F5	6.60 × $10^{02}$	4.15 × $10^{01}$	6.27 × $10^{02}$	3.38 × $10^{01}$	6.56 × $10^{02}$	4.48 × $10^{01}$	**6.27 × $10^{02}$**	**3.12 × $10^{01}$**
F6	6.28 × $10^{02}$	9.84 × $10^{00}$	**6.20 × $10^{02}$**	**7.85 × $10^{00}$**	6.32 × $10^{02}$	1.05 × $10^{01}$	6.26 × $10^{02}$	9.15 × $10^{00}$
F7	8.69 × $10^{02}$	3.44 × $10^{01}$	**8.53 × $10^{02}$**	**3.28 × $10^{01}$**	9.08 × $10^{02}$	6.10 × $10^{01}$	9.05 × $10^{02}$	7.25 × $10^{01}$
F8	9.23 × $10^{02}$	3.15 × $10^{01}$	9.20 × $10^{02}$	2.85 × $10^{01}$	9.13 × $10^{02}$	2.87 × $10^{01}$	**8.08 × $10^{02}$**	**2.15 × $10^{01}$**
F9	2.89 × $10^{03}$	1.25 × $10^{03}$	**1.70 × $10^{03}$**	**7.54 × $10^{02}$**	2.45 × $10^{03}$	8.51 × $10^{02}$	1.84 × $10^{03}$	6.53 × $10^{02}$
F10	5.13 × $10^{03}$	6.63 × $10^{02}$	5.28 × $10^{03}$	6.53 × $10^{02}$	**5.06 × $10^{03}$**	**5.47 × $10^{02}$**	5.10 × $10^{03}$	5.56 × $10^{02}$
F11	1.29 × $10^{03}$	5.17 × $10^{01}$	1.29 × $10^{03}$	5.46 × $10^{01}$	1.27 × $10^{03}$	5.65 × $10^{01}$	**1.27 × $10^{03}$**	**4.44 × $10^{01}$**
F12	9.80 × $10^{06}$	9.35 × $10^{06}$	1.11 × $10^{07}$	9.47 × $10^{06}$	**5.97 × $10^{06}$**	**4.30 × $10^{06}$**	8.15 × $10^{06}$	5.86 × $10^{06}$
F13	2.62 × $10^{05}$	3.10 × $10^{05}$	1.15 × $10^{05}$	1.23 × $10^{05}$	2.12 × $10^{05}$	6.41 × $10^{05}$	**8.97 × $10^{04}$**	**7.98 × $10^{04}$**
F14	4.69 × $10^{04}$	4.69 × $10^{04}$	**3.31 × $10^{04}$**	**2.86 × $10^{04}$**	4.29 × $10^{04}$	4.67 × $10^{04}$	3.45 × $10^{04}$	3.24 × $10^{04}$
F15	6.24 × $10^{04}$	6.16 × $10^{04}$	2.02 × $10^{04}$	2.39 × $10^{04}$	4.19 × $10^{04}$	4.53 × $10^{04}$	**1.59 × $10^{04}$**	**1.25 × $10^{04}$**
F16	2.80 × $10^{03}$	3.27 × $10^{02}$	2.79 × $10^{03}$	2.95 × $10^{02}$	2.81 × $10^{03}$	3.46 × $10^{02}$	**2.78 × $10^{03}$**	**3.85 × $10^{02}$**
F17	2.30 × $10^{03}$	2.48 × $10^{02}$	**2.15 × $10^{03}$**	**1.96 × $10^{02}$**	2.32 × $10^{03}$	2.15 × $10^{02}$	2.25 × $10^{03}$	2.25 × $10^{02}$
F18	2.84 × $10^{05}$	1.99 × $10^{05}$	**1.99 × $10^{05}$**	**1.24 × $10^{05}$**	3.19 × $10^{05}$	2.85 × $10^{05}$	2.84 × $10^{05}$	2.56 × $10^{05}$
F19	8.13 × $10^{04}$	9.96 × $10^{04}$	3.47 × $10^{04}$	5.84 × $10^{04}$	8.36 × $10^{04}$	1.40 × $10^{05}$	**3.63 × $10^{04}$**	**4.26 × $10^{04}$**
F20	2.53 × $10^{03}$	1.87 × $10^{02}$	**2.49 × $10^{03}$**	**1.84 × $10^{02}$**	2.54 × $10^{03}$	1.95 × $10^{02}$	2.51 × $10^{03}$	1.96 × $10^{02}$
F21	2.42 × $10^{03}$	2.31 × $10^{01}$	2.40 × $10^{03}$	2.50 × $10^{01}$	2.43 × $10^{03}$	3.19 × $10^{01}$	**2.42 × $10^{03}$**	**2.74 × $10^{01}$**
F22	2.66 × $10^{03}$	1.27 × $10^{03}$	**2.45 × $10^{03}$**	**5.91 × $10^{02}$**	3.15 × $10^{03}$	1.75 × $10^{03}$	2.71 × $10^{03}$	1.19 × $10^{03}$
F23	2.80 × $10^{03}$	5.20 × $10^{01}$	**2.77 × $10^{03}$**	**2.64 × $10^{01}$**	2.83 × $10^{03}$	5.62 × $10^{01}$	2.82 × $10^{03}$	5.02 × $10^{01}$
F24	2.97 × $10^{03}$	3.86 × $10^{01}$	2.94 × $10^{03}$	2.94 × $10^{01}$	3.00 × $10^{03}$	5.86 × $10^{01}$	**2.98 × $10^{03}$**	**4.54 × $10^{01}$**
F25	2.91 × $10^{03}$	2.00 × $10^{01}$	2.92 × $10^{03}$	2.59 × $10^{01}$	**2.90 × $10^{03}$**	**1.99 × $10^{01}$**	2.91 × $10^{03}$	2.55 × $10^{01}$
F26	4.87 × $10^{03}$	1.05 × $10^{03}$	**4.36 × $10^{03}$**	**9.53 × $10^{02}$**	5.98 × $10^{03}$	9.41 × $10^{02}$	5.46 × $10^{03}$	7.28 × $10^{02}$
F27	3.20 × $10^{03}$	3.25 × $10^{-04}$	3.20 × $10^{03}$	2.91 × $10^{-04}$	3.20 × $10^{03}$	2.82 × $10^{-04}$	**3.20 × $10^{03}$**	**2.35 × $10^{-04}$**
F28	3.30 × $10^{03}$	5.44 × $10^{00}$	3.29 × $10^{03}$	5.09 × $10^{00}$	3.30 × $10^{03}$	5.55 × $10^{00}$	**3.29 × $10^{03}$**	**5.72 × $10^{00}$**
F29	3.91 × $10^{03}$	2.97 × $10^{02}$	3.72 × $10^{03}$	2.31 × $10^{02}$	3.88 × $10^{03}$	2.37 × $10^{02}$	**3.79 × $10^{03}$**	**2.50 × $10^{02}$**
F30	**1.09 × $10^{05}$**	**7.77 × $10^{04}$**	1.76 × $10^{05}$	2.83 × $10^{05}$	8.69 × $10^{04}$	1.25 × $10^{05}$	7.97 × $10^{06}$	9.72 × $10^{06}$
**Win**	**1**		**10**		**4**		**14**	

**Table 5:** Use the test results of CEC2017 on 50 dimensions and compare with the original algorithm and other improvements of the original algorithm (bold font indicates optimal data).

Function	BFGO		QBFGO		OBFGO		OQBFGO	
	Mean	Std	Mean	Std	Mean	Std	Mean	Std
F1	3.52 × $10^{09}$	5.62 × $10^{09}$	3.75 × $10^{07}$	5.12 × $10^{07}$	**4.91 × $10^{06}$**	**6.01 × $10^{06}$**	5.97 × $10^{08}$	3.60 × $10^{08}$
F3	**3.82 × $10^{04}$**	**3.59 × $10^{04}$**	5.71 × $10^{04}$	9.29 × $10^{03}$	8.23 × $10^{04}$	1.35 × $10^{04}$	6.56 × $10^{04}$	1.17 × $10^{04}$
F4	1.18 × $10^{03}$	6.48 × $10^{02}$	6.01 × $10^{02}$	4.72 × $10^{01}$	**5.79 × $10^{02}$**	**5.64 × $10^{01}$**	7.41 × $10^{02}$	1.07 × $10^{02}$
F5	8.84 × $10^{02}$	4.81 × $10^{01}$	7.77 × $10^{02}$	3.49 × $10^{01}$	**7.58 × $10^{02}$**	**4.82 × $10^{01}$**	8.11 × $10^{02}$	3.96 × $10^{01}$
F6	6.60 × $10^{02}$	8.65 × $10^{00}$	6.39 × $10^{02}$	9.95 × $10^{00}$	6.43 × $10^{02}$	9.40 × $10^{00}$	**6.35 × $10^{02}$**	**9.14 × $10^{00}$**
F7	1.52 × $10^{03}$	1.14 × $10^{02}$	1.19 × $10^{03}$	9.58 × $10^{01}$	1.23 × $10^{03}$	8.01 × $10^{01}$	**1.09 × $10^{03}$**	**7.14 × $10^{01}$**
F8	1.17 × $10^{03}$	4.87 × $10^{01}$	**1.07 × $10^{03}$**	**5.67 × $10^{01}$**	1.07 × $10^{03}$	5.70 × $10^{01}$	1.11 × $10^{03}$	5.32 × $10^{01}$
F9	1.18 × $10^{04}$	2.95 × $10^{03}$	7.69 × $10^{03}$	2.84 × $10^{03}$	1.01 × $10^{04}$	2.83 × $10^{03}$	**7.63 × $10^{03}$**	**4.39 × $10^{03}$**
F10	9.68 × $10^{03}$	1.00 × $10^{03}$	**8.23 × $10^{03}$**	**1.16 × $10^{03}$**	8.47 × $10^{03}$	1.03 × $10^{03}$	8.24 × $10^{03}$	1.05 × $10^{03}$
F11	1.70 × $10^{03}$	1.90 × $10^{02}$	1.52 × $10^{03}$	1.20 × $10^{02}$	1.55 × $10^{03}$	1.19 × $10^{02}$	**1.53 × $10^{03}$**	**7.80 × $10^{01}$**
F12	2.59 × $10^{09}$	4.27 × $10^{09}$	5.40 × $10^{07}$	3.55 × $10^{07}$	**3.87 × $10^{07}$**	**2.52 × $10^{07}$**	9.40 × $10^{07}$	6.05 × $10^{07}$
F13	1.06 × $10^{09}$	2.49 × $10^{09}$	2.23 × $10^{06}$	5.08 × $10^{06}$	**1.17 × $10^{06}$**	**3.68 × $10^{06}$**	1.81 × $10^{06}$	2.02 × $10^{06}$
F14	1.12 × $10^{06}$	2.85 × $10^{06}$	3.61 × $10^{05}$	2.75 × $10^{05}$	3.94 × $10^{05}$	2.52 × $10^{05}$	**3.19 × $10^{05}$**	**2.51 × $10^{05}$**
F15	7.62 × $10^{07}$	7.70 × $10^{07}$	6.74 × $10^{04}$	6.84 × $10^{04}$	**4.92 × $10^{04}$**	**4.13 × $10^{04}$**	6.63 × $10^{04}$	4.84 × $10^{04}$
F16	4.08 × $10^{03}$	6.18 × $10^{02}$	3.67 × $10^{03}$	5.23 × $10^{02}$	3.75 × $10^{03}$	5.31 × $10^{02}$	**3.61 × $10^{03}$**	**4.04 × $10^{02}$**
F17	3.86 × $10^{03}$	4.70 × $10^{02}$	3.38 × $10^{03}$	3.43 × $10^{02}$	3.45 × $10^{03}$	3.49 × $10^{02}$	**3.14 × $10^{03}$**	**2.92 × $10^{02}$**
F18	4.34 × $10^{06}$	1.60 × $10^{07}$	2.03 × $10^{06}$	1.37 × $10^{06}$	2.06 × $10^{06}$	1.65 × $10^{06}$	**1.68 × $10^{06}$**	**1.06 × $10^{06}$**
F19	7.29 × $10^{05}$	3.30 × $10^{07}$	1.61 × $10^{05}$	2.06 × $10^{05}$	2.30 × $10^{05}$	5.31 × $10^{05}$	**1.50 × $10^{05}$**	**1.74 × $10^{05}$**
F20	3.40 × $10^{03}$	3.18 × $10^{02}$	3.19 × $10^{03}$	3.15 × $10^{02}$	3.11 × $10^{03}$	3.88 × $10^{02}$	**3.17 × $10^{03}$**	**2.85 × $10^{02}$**
F21	2.71 × $10^{03}$	6.56 × $10^{01}$	2.55 × $10^{03}$	6.22 × $10^{01}$	2.56 × $10^{03}$	5.52 × $10^{01}$	**2.55 × $10^{03}$**	**4.53 × $10^{01}$**
F22	**1.10 × $10^{04}$**	**9.48 × $10^{02}$**	1.03 × $10^{04}$	1.79 × $10^{03}$	9.95 × $10^{03}$	1.75 × $10^{03}$	1.04 × $10^{04}$	1.09 × $10^{03}$
F23	3.46 × $10^{03}$	1.40 × $10^{02}$	3.08 × $10^{03}$	1.09 × $10^{02}$	3.16 × $10^{03}$	1.41 × $10^{02}$	**3.01 × $10^{03}$**	**6.78 × $10^{01}$**
F24	3.70 × $10^{03}$	1.74 × $10^{02}$	3.25 × $10^{03}$	8.42 × $10^{01}$	3.35 × $10^{03}$	9.78 × $10^{01}$	**3.20 × $10^{03}$**	**5.15 × $10^{01}$**
F25	3.41 × $10^{03}$	1.35 × $10^{02}$	3.05 × $10^{03}$	4.20 × $10^{01}$	**3.00 × $10^{03}$**	**4.25 × $10^{01}$**	3.18 × $10^{03}$	7.23 × $10^{01}$
F26	1.08 × $10^{04}$	1.13 × $10^{03}$	8.87 × $10^{03}$	1.90 × $10^{03}$	8.54 × $10^{03}$	1.64 × $10^{03}$	**5.16 × $10^{03}$**	**1.90 × $10^{03}$**
F27	3.98 × $10^{03}$	2.11 × $10^{02}$	**3.20 × $10^{03}$**	**2.54 × $10^{-04}$**	3.20 × $10^{03}$	3.01 × $10^{-04}$	3.20 × $10^{03}$	3.25 × $10^{-04}$
F28	4.41 × $10^{03}$	1.32 × $10^{03}$	3.30 × $10^{03}$	8.89 × $10^{00}$	3.30 × $10^{03}$	7.16 × $10^{00}$	**3.29 × $10^{03}$**	**9.13 × $10^{00}$**
F29	6.41 × $10^{03}$	1.16 × $10^{03}$	**4.56 × $10^{03}$**	**4.40 × $10^{02}$**	4.86 × $10^{03}$	6.00 × $10^{02}$	4.59 × $10^{03}$	5.44 × $10^{02}$
F30	3.06 × $10^{07}$	9.05 × $10^{07}$	**9.57 × $10^{05}$**	**9.24 × $10^{05}$**	9.97 × $10^{05}$	1.36 × $10^{06}$	9.94 × $10^{05}$	9.39 × $10^{05}$
**Win**	**2**		**5**		**7**		**15**	

**Table 6:** Use the test results of CEC2017 on 10 dimensions and compare with mature algorithms (bold font indicates optimal data).

Function	OQBFGO		PSO		GWO		DE	
	Mean	Std	Mean	Std	Mean	Std	Mean	Std
F1	**9.76 × $10^{03}$**	**1.28 × $10^{04}$**	1.05 × $10^{08}$	4.04 × $10^{08}$	5.58 × $10^{07}$	2.93 × $10^{08}$	9.49 × $10^{08}$	3.83 × $10^{08}$
F3	2.35 × $10^{02}$	4.09 × $10^{01}$	**1.07 × $10^{07}$**	**5.52 × $10^{07}$**	2.45 × $10^{08}$	6.99 × $10^{08}$	3.60 × $10^{08}$	1.03 × $10^{09}$
F4	**4.10 × $10^{02}$**	**1.72 × $10^{01}$**	4.12 × $10^{02}$	1.93 × $10^{01}$	4.12 × $10^{02}$	1.91 × $10^{01}$	4.84 × $10^{02}$	3.03 × $10^{01}$
F5	**5.15 × $10^{02}$**	**5.23 × $10^{00}$**	5.27 × $10^{02}$	1.10 × $10^{01}$	5.31 × $10^{02}$	1.07 × $10^{01}$	5.53 × $10^{02}$	7.69 × $10^{00}$
F6	**6.01 × $10^{02}$**	**1.01 × $10^{00}$**	6.06 × $10^{02}$	4.06 × $10^{00}$	6.05 × $10^{02}$	4.09 × $10^{00}$	6.31 × $10^{02}$	4.57 × $10^{00}$
F7	**7.27 × $10^{02}$**	**7.67 × $10^{00}$**	7.38 × $10^{02}$	1.37 × $10^{01}$	7.34 × $10^{02}$	1.04 × $10^{01}$	9.72 × $10^{02}$	3.85 × $10^{01}$
F8	**8.16 × $10^{02}$**	**5.45 × $10^{00}$**	8.23 × $10^{02}$	1.06 × $10^{01}$	8.24 × $10^{02}$	8.98 × $10^{00}$	8.82 × $10^{02}$	8.93 × $10^{00}$
F9	**9.01 × $10^{02}$**	**1.23 × $10^{00}$**	9.26 × $10^{02}$	4.42 × $10^{01}$	9.10 × $10^{02}$	1.28 × $10^{01}$	2.84 × $10^{03}$	3.82 × $10^{02}$
F10	**1.68 × $10^{03}$**	**3.54 × $10^{02}$**	1.81 × $10^{03}$	2.42 × $10^{02}$	1.84 × $10^{03}$	3.14 × $10^{02}$	1.80 × $10^{03}$	1.96 × $10^{02}$
F11	**1.13 × $10^{03}$**	**1.53 × $10^{01}$**	1.17 × $10^{03}$	5.60 × $10^{01}$	1.17 × $10^{03}$	8.01 × $10^{01}$	1.44 × $10^{03}$	1.91 × $10^{02}$
F12	**1.41 × $10^{04}$**	**1.17 × $10^{04}$**	2.06 × $10^{06}$	5.03 × $10^{06}$	9.89 × $10^{05}$	2.60 × $10^{06}$	6.87 × $10^{07}$	1.48 × $10^{08}$
F13	**4.04 × $10^{03}$**	**3.80 × $10^{03}$**	5.18 × $10^{03}$	1.34 × $10^{04}$	8.07 × $10^{03}$	1.39 × $10^{04}$	1.66 × $10^{03}$	8.57 × $10^{01}$
F14	**1.43 × $10^{03}$**	**9.94 × $10^{00}$**	1.47 × $10^{03}$	3.22 × $10^{01}$	1.48 × $10^{03}$	4.74 × $10^{01}$	1.45 × $10^{03}$	1.56 × $10^{01}$
F15	**1.53 × $10^{03}$**	**1.88 × $10^{01}$**	1.58 × $10^{03}$	7.02 × $10^{01}$	1.82 × $10^{03}$	1.04 × $10^{03}$	1.66 × $10^{03}$	1.08 × $10^{02}$
F16	**1.67 × $10^{03}$**	**6.66 × $10^{01}$**	1.68 × $10^{03}$	7.48 × $10^{01}$	1.72 × $10^{03}$	1.32 × $10^{02}$	1.79 × $10^{03}$	8.52 × $10^{01}$
F17	**1.74 × $10^{03}$**	**1.80 × $10^{01}$**	1.77 × $10^{03}$	2.58 × $10^{01}$	1.76 × $10^{03}$	2.71 × $10^{01}$	1.77 × $10^{03}$	8.16 × $10^{00}$
F18	9.67 × $10^{03}$	8.38 × $10^{03}$	1.82 × $10^{04}$	1.63 × $10^{04}$	1.76 × $10^{04}$	1.60 × $10^{04}$	**2.12 × $10^{03}$**	**1.09 × $10^{02}$**
F19	**1.91 × $10^{03}$**	**8.89 × $10^{00}$**	1.97 × $10^{03}$	1.43 × $10^{02}$	1.95 × $10^{03}$	5.39 × $10^{01}$	1.96 × $10^{03}$	4.17 × $10^{01}$
F20	**2.04 × $10^{03}$**	**3.34 × $10^{01}$**	2.06 × $10^{03}$	4.26 × $10^{01}$	2.09 × $10^{03}$	6.18 × $10^{01}$	2.05 × $10^{03}$	1.02 × $10^{01}$
F21	**2.21 × $10^{03}$**	**3.56 × $10^{01}$**	2.30 × $10^{03}$	5.35 × $10^{01}$	2.28 × $10^{03}$	6.04 × $10^{01}$	2.28 × $10^{03}$	5.56 × $10^{01}$
F22	2.30 × $10^{03}$	2.52 × $10^{01}$	**2.32 × $10^{03}$**	**1.65 × $10^{01}$**	2.31 × $10^{03}$	1.98 × $10^{01}$	2.49 × $10^{03}$	2.17 × $10^{02}$
F23	**2.62 × $10^{03}$**	**7.99 × $10^{00}$**	2.64 × $10^{03}$	1.24 × $10^{01}$	2.64 × $10^{03}$	1.09 × $10^{01}$	2.63 × $10^{03}$	8.83 × $10^{00}$
F24	2.63 × $10^{03}$	1.27 × $10^{02}$	**2.75 × $10^{03}$**	**6.96 × $10^{01}$**	2.73 × $10^{03}$	9.24 × $10^{01}$	2.77 × $10^{03}$	8.33 × $10^{00}$
F25	**2.93 × $10^{03}$**	**2.25 × $10^{01}$**	2.94 × $10^{03}$	3.71 × $10^{01}$	2.93 × $10^{03}$	3.20 × $10^{01}$	3.02 × $10^{03}$	1.46 × $10^{01}$
F26	**2.95 × $10^{03}$**	**7.80 × $10^{01}$**	3.15 × $10^{03}$	3.62 × $10^{02}$	3.05 × $10^{03}$	2.18 × $10^{02}$	3.13 × $10^{03}$	2.23 × $10^{02}$
F27	**3.08 × $10^{03}$**	**2.30 × $10^{01}$**	3.12 × $10^{03}$	2.90 × $10^{01}$	3.11 × $10^{03}$	1.21 × $10^{01}$	3.10 × $10^{03}$	3.00 × $10^{00}$
F28	**3.26 × $10^{03}$**	**4.03 × $10^{01}$**	3.37 × $10^{03}$	1.08 × $10^{02}$	3.37 × $10^{03}$	8.99 × $10^{01}$	3.27 × $10^{03}$	5.44 × $10^{01}$
F29	**3.18 × $10^{03}$**	**2.63 × $10^{01}$**	3.22 × $10^{03}$	4.97 × $10^{01}$	3.22 × $10^{03}$	5.87 × $10^{01}$	3.19 × $10^{03}$	3.34 × $10^{01}$
F30	**3.41 × $10^{03}$**	**2.62 × $10^{02}$**	1.01 × $10^{06}$	1.31 × $10^{06}$	1.90 × $10^{06}$	3.34 × $10^{06}$	8.56 × $10^{05}$	1.09 × $10^{05}$
**Win**	**25**		**3**		**0**		**1**	

**Table 7:** Use the test results of CEC2017 on 30 dimensions and compare with mature algorithms (bold font indicates optimal data).

Function	OQBFGO		PSO		GWO		DE	
	Mean	Std	Mean	Std	Mean	Std	Mean	Std
F1	**7.97 × $10^{06}$**	**9.72 × $10^{06}$**	1.56 × $10^{09}$	2.18 × $10^{09}$	1.11 × $10^{09}$	1.01 × $10^{09}$	7.31 × $10^{10}$	6.55 × $10^{09}$
F3	**4.22 × $10^{03}$**	**1.78 × $10^{03}$**	1.14 × $10^{04}$	5.37 × $10^{03}$	3.94 × $10^{04}$	1.04 × $10^{04}$	3.00 × $10^{05}$	4.31 × $10^{04}$
F4	**4.97 × $10^{02}$**	**2.30 × $10^{01}$**	7.01 × $10^{02}$	2.45 × $10^{02}$	5.58 × $10^{02}$	3.91 × $10^{01}$	7.42 × $10^{03}$	1.09 × $10^{03}$
F5	6.27 × $10^{02}$	3.12 × $10^{01}$	6.71 × $10^{02}$	4.72 × $10^{01}$	**5.98 × $10^{02}$**	**4.80 × $10^{01}$**	1.01 × $10^{03}$	1.98 × $10^{01}$
F6	6.26 × $10^{02}$	9.15 × $10^{00}$	6.42 × $10^{02}$	1.05 × $10^{01}$	**6.05 × $10^{02}$**	**2.21 × $10^{00}$**	6.87 × $10^{02}$	4.12 × $10^{00}$
F7	9.05 × $10^{02}$	7.25 × $10^{01}$	1.04 × $10^{03}$	7.91 × $10^{01}$	**8.57 × $10^{02}$**	**4.96 × $10^{01}$**	3.30 × $10^{03}$	1.76 × $10^{02}$
F8	**8.08 × $10^{02}$**	**2.15 × $10^{01}$**	9.48 × $10^{02}$	2.68 × $10^{01}$	8.83 × $10^{02}$	2.97 × $10^{01}$	1.28 × $10^{03}$	2.37 × $10^{01}$
F9	**1.84 × $10^{03}$**	**6.53 × $10^{02}$**	4.19 × $10^{03}$	1.50 × $10^{03}$	1.56 × $10^{03}$	7.83 × $10^{02}$	2.47 × $10^{04}$	3.17 × $10^{03}$
F10	**5.10 × $10^{03}$**	**5.56 × $10^{02}$**	5.44 × $10^{03}$	7.31 × $10^{02}$	4.36 × $10^{03}$	1.03 × $10^{03}$	6.53 × $10^{03}$	3.92 × $10^{02}$
F11	**1.27 × $10^{03}$**	**4.44 × $10^{01}$**	1.40 × $10^{03}$	1.10 × $10^{02}$	1.47 × $10^{03}$	2.90 × $10^{02}$	6.66 × $10^{03}$	3.11 × $10^{03}$
F12	**8.15 × $10^{06}$**	**5.86 × $10^{06}$**	2.02 × $10^{08}$	5.98 × $10^{08}$	3.06 × $10^{07}$	3.10 × $10^{07}$	4.66 × $10^{09}$	1.02 × $10^{09}$
F13	**8.97 × $10^{04}$**	**7.98 × $10^{04}$**	1.05 × $10^{07}$	2.45 × $10^{07}$	9.12 × $10^{05}$	4.30 × $10^{06}$	5.59 × $10^{08}$	3.15 × $10^{08}$
F14	**3.45 × $10^{04}$**	**3.24 × $10^{04}$**	3.81 × $10^{04}$	7.94 × $10^{04}$	1.40 × $10^{05}$	2.23 × $10^{05}$	2.50 × $10^{05}$	3.74 × $10^{05}$
F15	1.59 × $10^{04}$	1.25 × $10^{04}$	**1.21 × $10^{04}$**	**1.46 × $10^{04}$**	4.37 × $10^{05}$	9.85 × $10^{05}$	7.16 × $10^{07}$	2.38 × $10^{08}$
F16	2.78 × $10^{03}$	3.85 × $10^{02}$	2.81 × $10^{03}$	3.20 × $10^{02}$	**2.46 × $10^{03}$**	**3.19 × $10^{02}$**	3.67 × $10^{03}$	2.57 × $10^{02}$
F17	2.25 × $10^{03}$	2.25 × $10^{02}$	2.32 × $10^{03}$	2.54 × $10^{02}$	**2.01 × $10^{03}$**	**1.63 × $10^{02}$**	3.02 × $10^{03}$	1.58 × $10^{02}$
F18	**2.84 × $10^{05}$**	**2.56 × $10^{05}$**	8.65 × $10^{05}$	2.83 × $10^{06}$	1.06 × $10^{06}$	1.51 × $10^{06}$	1.20 × $10^{07}$	1.82 × $10^{07}$
F19	**3.63 × $10^{04}$**	**4.26 × $10^{04}$**	2.20 × $10^{06}$	9.09 × $10^{06}$	8.50 × $10^{05}$	1.58 × $10^{06}$	1.14 × $10^{08}$	3.39 × $10^{07}$
F20	2.51 × $10^{03}$	1.96 × $10^{02}$	2.58 × $10^{03}$	1.81 × $10^{02}$	**2.35 × $10^{03}$**	**1.26 × $10^{02}$**	2.86 × $10^{03}$	1.61 × $10^{02}$
F21	2.42 × $10^{03}$	2.74 × $10^{01}$	2.46 × $10^{03}$	3.84 × $10^{01}$	**2.37 × $10^{03}$**	**1.65 × $10^{01}$**	2.76 × $10^{03}$	2.22 × $10^{01}$
F22	**2.71 × $10^{03}$**	**1.19 × $10^{03}$**	4.95 × $10^{03}$	2.07 × $10^{03}$	5.01 × $10^{03}$	1.77 × $10^{03}$	8.05 × $10^{03}$	6.19 × $10^{02}$
F23	2.82 × $10^{03}$	5.02 × $10^{01}$	2.96 × $10^{03}$	7.27 × $10^{01}$	**2.74 × $10^{03}$**	**3.18 × $10^{01}$**	3.05 × $10^{03}$	2.01 × $10^{01}$
F24	**2.98 × $10^{03}$**	**4.54 × $10^{01}$**	3.14 × $10^{03}$	5.77 × $10^{01}$	2.93 × $10^{03}$	6.33 × $10^{01}$	3.13 × $10^{03}$	1.52 × $10^{01}$
F25	**2.91 × $10^{03}$**	**2.55 × $10^{01}$**	2.95 × $10^{03}$	5.92 × $10^{01}$	2.96 × $10^{03}$	3.48 × $10^{01}$	9.12 × $10^{03}$	5.79 × $10^{02}$
F26	5.46 × $10^{03}$	7.28 × $10^{02}$	6.51 × $10^{03}$	9.17 × $10^{02}$	**4.39 × $10^{03}$**	**3.34 × $10^{02}$**	8.14 × $10^{03}$	2.15 × $10^{02}$
F27	**3.20 × $10^{03}$**	**2.35 × $10^{-04}$**	3.34 × $10^{03}$	6.66 × $10^{01}$	3.24 × $10^{03}$	2.06 × $10^{01}$	3.30 × $10^{03}$	2.88 × $10^{01}$
F28	**3.29 × $10^{03}$**	**5.72 × $10^{00}$**	3.53 × $10^{03}$	3.21 × $10^{02}$	3.37 × $10^{03}$	6.73 × $10^{01}$	6.49 × $10^{03}$	7.76 × $10^{01}$
F29	3.79 × $10^{03}$	2.50 × $10^{02}$	4.48 × $10^{03}$	4.31 × $10^{02}$	**3.73 × $10^{03}$**	**1.33 × $10^{02}$**	4.50 × $10^{03}$	2.98 × $10^{02}$
F30	**1.77 × $10^{05}$**	**2.55 × $10^{05}$**	5.50 × $10^{05}$	8.56 × $10^{05}$	4.91 × $10^{06}$	3.16 × $10^{06}$	8.97 × $10^{07}$	2.95 × $10^{07}$
**Win**	**18**		**1**		**10**		**0**	

**Table 8:** Use the test results of CEC2017 on 50 dimensions and compare with mature algorithms (bold font indicates optimal data).

Function	OQBFGO		PSO		GWO		DE	
	Mean	Std	Mean	Std	Mean	Std	Mean	Std
F1	**5.97 × $10^{08}$**	**3.60 × $10^{08}$**	3.52 × $10^{09}$	6.26 × $10^{09}$	6.12 × $10^{09}$	2.84 × $10^{09}$	1.63 × $10^{11}$	1.02 × $10^{10}$
F3	**6.56 × $10^{04}$**	**1.17 × $10^{04}$**	3.82 × $10^{04}$	1.23 × $10^{04}$	9.31 × $10^{04}$	1.79 × $10^{04}$	5.64 × $10^{05}$	9.02 × $10^{04}$
F4	**7.41 × $10^{02}$**	**1.07 × $10^{02}$**	1.18 × $10^{03}$	7.82 × $10^{02}$	1.01 × $10^{03}$	4.32 × $10^{02}$	3.00 × $10^{04}$	5.19 × $10^{03}$
F5	8.11 × $10^{02}$	3.96 × $10^{01}$	8.84 × $10^{02}$	5.14 × $10^{01}$	**6.97 × $10^{02}$**	**5.00 × $10^{01}$**	1.47 × $10^{03}$	3.32 × $10^{01}$
F6	6.35 × $10^{02}$	9.14 × $10^{00}$	6.60 × $10^{02}$	5.99 × $10^{00}$	**6.13 × $10^{02}$**	**3.87 × $10^{00}$**	7.10 × $10^{02}$	3.80 × $10^{00}$
F7	**1.09 × $10^{03}$**	**7.14 × $10^{01}$**	1.52 × $10^{03}$	1.59 × $10^{02}$	1.12 × $10^{03}$	4.41 × $10^{01}$	5.89 × $10^{03}$	3.78 × $10^{02}$
F8	**1.11 × $10^{03}$**	**5.32 × $10^{01}$**	1.17 × $10^{03}$	5.85 × $10^{01}$	1.21 × $10^{03}$	4.90 × $10^{01}$	1.77 × $10^{03}$	3.58 × $10^{01}$
F9	7.63 × $10^{03}$	4.39 × $10^{03}$	1.18 × $10^{04}$	1.96 × $10^{03}$	**5.35 × $10^{03}$**	**2.99 × $10^{03}$**	5.87 × $10^{04}$	6.91 × $10^{03}$
F10	8.24 × $10^{03}$	1.05 × $10^{03}$	9.68 × $10^{03}$	1.07 × $10^{03}$	**6.87 × $10^{03}$**	**1.66 × $10^{03}$**	1.10 × $10^{04}$	6.44 × $10^{02}$
F11	**1.53 × $10^{03}$**	**7.80 × $10^{01}$**	1.70 × $10^{03}$	3.29 × $10^{02}$	3.83 × $10^{03}$	1.83 × $10^{03}$	2.90 × $10^{04}$	1.18 × $10^{04}$
F12	**9.40 × $10^{07}$**	**6.05 × $10^{07}$**	2.59 × $10^{09}$	3.29 × $10^{09}$	4.66 × $10^{08}$	5.90 × $10^{08}$	3.53 × $10^{10}$	4.66 × $10^{09}$
F13	**1.81 × $10^{06}$**	**2.02 × $10^{06}$**	1.06 × $10^{09}$	1.67 × $10^{09}$	1.65 × $10^{08}$	2.05 × $10^{08}$	8.39 × $10^{09}$	8.46 × $10^{08}$
F14	**3.19 × $10^{05}$**	**2.51 × $10^{05}$**	1.12 × $10^{06}$	2.48 × $10^{06}$	7.11 × $10^{05}$	1.24 × $10^{06}$	4.84 × $10^{06}$	5.26 × $10^{06}$
F15	**6.63 × $10^{04}$**	**4.84 × $10^{04}$**	7.62 × $10^{07}$	2.95 × $10^{08}$	1.07 × $10^{07}$	1.73 × $10^{07}$	8.98 × $10^{08}$	4.56 × $10^{08}$
F16	3.61 × $10^{03}$	4.04 × $10^{02}$	4.08 × $10^{03}$	4.33 × $10^{02}$	**3.09 × $10^{03}$**	**4.52 × $10^{02}$**	7.31 × $10^{03}$	3.13 × $10^{02}$
F17	3.14 × $10^{03}$	2.92 × $10^{02}$	3.86 × $10^{03}$	3.97 × $10^{02}$	**2.79 × $10^{03}$**	**3.50 × $10^{02}$**	2.37 × $10^{04}$	1.54 × $10^{04}$
F18	**1.68 × $10^{06}$**	**1.06 × $10^{06}$**	4.34 × $10^{06}$	1.07 × $10^{07}$	3.29 × $10^{06}$	2.30 × $10^{06}$	2.93 × $10^{07}$	2.56 × $10^{07}$
F19	**1.50 × $10^{05}$**	**1.74 × $10^{05}$**	7.29 × $10^{05}$	2.02 × $10^{06}$	2.76 × $10^{06}$	6.18 × $10^{06}$	6.19 × $10^{08}$	1.89 × $10^{08}$
F20	3.17 × $10^{03}$	2.85 × $10^{02}$	3.40 × $10^{03}$	3.08 × $10^{02}$	**2.82 × $10^{03}$**	**2.98 × $10^{02}$**	3.94 × $10^{03}$	1.66 × $10^{02}$
F21	2.55 × $10^{03}$	4.53 × $10^{01}$	2.71 × $10^{03}$	5.62 × $10^{01}$	**2.50 × $10^{03}$**	**5.83 × $10^{01}$**	3.25 × $10^{03}$	4.89 × $10^{01}$
F22	1.04 × $10^{04}$	1.09 × $10^{03}$	1.10 × $10^{04}$	1.10 × $10^{03}$	**9.55 × $10^{03}$**	**2.32 × $10^{03}$**	1.32 × $10^{04}$	9.33 × $10^{02}$
F23	3.01 × $10^{03}$	6.78 × $10^{01}$	3.46 × $10^{03}$	1.51 × $10^{02}$	**2.95 × $10^{03}$**	**7.88 × $10^{01}$**	3.60 × $10^{03}$	5.45 × $10^{01}$
F24	**3.20 × $10^{03}$**	**5.15 × $10^{01}$**	3.70 × $10^{03}$	1.59 × $10^{02}$	3.11 × $10^{03}$	9.47 × $10^{01}$	3.54 × $10^{03}$	2.03 × $10^{01}$
F25	**3.18 × $10^{03}$**	**7.23 × $10^{01}$**	3.41 × $10^{03}$	5.22 × $10^{02}$	3.40 × $10^{03}$	1.67 × $10^{02}$	3.26 × $10^{04}$	3.68 × $10^{03}$
F26	**5.16 × $10^{03}$**	**1.90 × $10^{03}$**	1.08 × $10^{04}$	1.20 × $10^{03}$	6.07 × $10^{03}$	5.88 × $10^{02}$	1.21 × $10^{04}$	4.49 × $10^{02}$
F27	**3.20 × $10^{03}$**	**3.25 × $10^{-04}$**	3.98 × $10^{03}$	2.45 × $10^{02}$	3.53 × $10^{03}$	6.29 × $10^{01}$	3.65 × $10^{03}$	4.97 × $10^{01}$
F28	**3.29 × $10^{03}$**	**9.13 × $10^{00}$**	4.41 × $10^{03}$	1.06 × $10^{03}$	4.09 × $10^{03}$	2.81 × $10^{02}$	8.90 × $10^{03}$	2.63 × $10^{02}$
F29	4.59 × $10^{03}$	5.44 × $10^{02}$	6.41 × $10^{03}$	1.15 × $10^{03}$	**4.39 × $10^{03}$**	**3.78 × $10^{02}$**	6.15 × $10^{03}$	3.42 × $10^{02}$
F30	**9.94 × $10^{05}$**	**9.39 × $10^{05}$**	3.06 × $10^{07}$	6.56 × $10^{07}$	9.97 × $10^{07}$	3.29 × $10^{07}$	8.08 × $10^{08}$	4.95 × $10^{08}$
**Win**	**18**		**0**		**11**		**0**	

**Table 9:** Use the test results of CEC2013 on 30 dimensions and compare with the original algorithm and other improvements of the original algorithm (bold font indicates optimal data).

Function	BFGO		QBFGO		OBFGO		OQBFGO	
	Mean	Std	Mean	Std	Mean	Std	Mean	Std
F1	5.16 × $10^{03}$	1.99 × $10^{03}$	1.59 × $10^{03}$	1.11 × $10^{03}$	−1.25 × $10^{03}$	2.20 × $10^{02}$	−1.34 × $10^{03}$	**7.10 × $10^{01}$**
F2	9.01 × $10^{07}$	3.25 × $10^{07}$	6.42 × $10^{07}$	2.49 × $10^{07}$	**4.76 × $10^{07}$**	**2.14 × $10^{07}$**	4.99 × $10^{07}$	1.62 × $10^{07}$
F3	9.81 × $10^{10}$	1.10 × $10^{11}$	4.15 × $10^{10}$	2.42 × $10^{10}$	**3.04 × $10^{10}$**	**1.42 × $10^{10}$**	3.88 × $10^{10}$	1.99 × $10^{10}$
F4	5.10 × $10^{04}$	6.69 × $10^{03}$	4.27 × $10^{04}$	6.79 × $10^{03}$	**4.09 × $10^{04}$**	**6.18 × $10^{03}$**	4.57 × $10^{04}$	6.35 × $10^{03}$
F5	1.05 × $10^{03}$	8.20 × $10^{02}$	−9.27 × $10^{01}$	2.37 × $10^{02}$	−8.31 × $10^{02}$	9.58 × $10^{01}$	−9.36 × $10^{02}$	**3.33 × $10^{01}$**
F6	−1.96 × $10^{02}$	2.52 × $10^{02}$	−5.52 × $10^{02}$	9.90 × $10^{01}$	−7.26 × $10^{02}$	4.63 × $10^{01}$	−7.69 × $10^{02}$	**4.29 × $10^{01}$**
F7	−5.65 × $10^{02}$	8.10 × $10^{01}$	−6.43 × $10^{02}$	3.56 × $10^{01}$	**−6.46 × $10^{02}$**	**3.85 × $10^{01}$**	−6.33 × $10^{02}$	6.65 × $10^{01}$
F8	−6.79 × $10^{02}$	7.58 × $10^{-02}$	−6.79 × $10^{02}$	5.54 × $10^{-02}$	**−6.79 × $10^{02}$**	**5.14 × $10^{-02}$**	−6.79 × $10^{02}$	6.21 × $10^{-02}$
F9	−5.63 × $10^{02}$	3.84 × $10^{00}$	−5.65 × $10^{02}$	3.31 × $10^{00}$	−5.65 × $10^{02}$	3.54 × $10^{00}$	**−5.66 × $10^{02}$**	**3.91 × $10^{00}$**
F10	6.95 × $10^{02}$	3.03 × $10^{02}$	1.65 × $10^{02}$	2.22 × $10^{02}$	−2.49 × $10^{02}$	1.52 × $10^{02}$	**−3.71 × $10^{02}$**	**5.19 × $10^{01}$**
F11	3.14 × $10^{01}$	9.43 × $10^{01}$	**−8.20 × $10^{01}$**	**5.11 × $10^{01}$**	−1.47 × $10^{02}$	7.08 × $10^{01}$	−1.33 × $10^{02}$	1.01 × $10^{02}$
F12	8.21 × $10^{01}$	8.54 × $10^{01}$	**5.06 × $10^{00}$**	**5.60 × $10^{01}$**	8.63 × $10^{00}$	6.92 × $10^{01}$	3.83 × $10^{01}$	7.19 × $10^{01}$
F13	2.29 × $10^{02}$	7.07 × $10^{01}$	**1.25 × $10^{02}$**	**5.00 × $10^{01}$**	1.49 × $10^{02}$	6.26 × $10^{01}$	1.75 × $10^{02}$	7.27 × $10^{01}$
F14	4.99 × $10^{03}$	8.74 × $10^{02}$	4.64 × $10^{03}$	6.43 × $10^{02}$	2.46 × $10^{03}$	5.87 × $10^{02}$	**2.36 × $10^{03}$**	**6.34 × $10^{02}$**
F15	6.26 × $10^{03}$	8.23 × $10^{02}$	6.35 × $10^{03}$	7.20 × $10^{02}$	5.87 × $10^{03}$	8.49 × $10^{02}$	**5.43 × $10^{03}$**	**7.59 × $10^{02}$**
F16	2.03 × $10^{02}$	6.28 × $10^{-01}$	**2.03 × $10^{02}$**	**5.70 × $10^{-01}$**	2.03 × $10^{02}$	7.44 × $10^{-01}$	2.03 × $10^{02}$	6.15 × $10^{-01}$
F17	7.87 × $10^{02}$	7.98 × $10^{01}$	7.11 × $10^{02}$	8.42 × $10^{01}$	5.63 × $10^{02}$	5.55 × $10^{01}$	**4.89 × $10^{02}$**	**4.85 × $10^{01}$**
F18	8.58 × $10^{02}$	1.10 × $10^{02}$	7.70 × $10^{02}$	7.60 × $10^{01}$	**7.46 × $10^{02}$**	**5.87 × $10^{01}$**	7.66 × $10^{02}$	6.62 × $10^{01}$
F19	2.84 × $10^{03}$	1.79 × $10^{03}$	1.14 × $10^{03}$	6.75 × $10^{02}$	5.41 × $10^{02}$	2.50 × $10^{01}$	**5.26 × $10^{02}$**	**1.29 × $10^{01}$**
F20	6.15 × $10^{02}$	4.02 × $10^{-01}$	6.14 × $10^{02}$	6.37 × $10^{-01}$	6.15 × $10^{02}$	5.04 × $10^{-01}$	**6.15 × $10^{02}$**	**3.54 × $10^{-01}$**
F21	2.43 × $10^{03}$	3.12 × $10^{02}$	2.11 × $10^{03}$	4.18 × $10^{02}$	1.45 × $10^{03}$	4.03 × $10^{02}$	**1.20 × $10^{03}$**	**1.77 × $10^{02}$**
F22	6.70 × $10^{03}$	7.92 × $10^{02}$	6.37 × $10^{03}$	6.39 × $10^{02}$	4.40 × $10^{03}$	1.08 × $10^{03}$	**4.30 × $10^{03}$**	**9.60 × $10^{02}$**
F23	8.36 × $10^{03}$	8.97 × $10^{02}$	8.50 × $10^{03}$	6.58 × $10^{02}$	**7.55 × $10^{03}$**	**9.38 × $10^{02}$**	7.61 × $10^{03}$	9.66 × $10^{02}$
F24	1.30 × $10^{03}$	9.08 × $10^{00}$	1.30 × $10^{03}$	6.44 × $10^{00}$	**1.29 × $10^{03}$**	**8.74 × $10^{00}$**	1.29 × $10^{03}$	9.32 × $10^{00}$
F25	1.40 × $10^{03}$	9.77 × $10^{00}$	1.40 × $10^{03}$	6.03 × $10^{00}$	**1.39 × $10^{03}$**	**7.17 × $10^{00}$**	1.39 × $10^{03}$	1.01 × $10^{01}$
F26	1.56 × $10^{03}$	7.12 × $10^{01}$	1.54 × $10^{03}$	8.35 × $10^{01}$	1.52 × $10^{03}$	9.32 × $10^{01}$	**1.49 × $10^{03}$**	**9.14 × $10^{01}$**
F27	2.56 × $10^{03}$	8.21 × $10^{01}$	2.54 × $10^{03}$	8.36 × $10^{01}$	**2.49 × $10^{03}$**	**9.14 × $10^{01}$**	2.51 × $10^{03}$	8.55 × $10^{01}$
F28	4.57 × $10^{03}$	6.89 × $10^{02}$	3.75 × $10^{03}$	7.30 × $10^{02}$	**3.69 × $10^{03}$**	**1.11 × $10^{03}$**	4.24 × $10^{03}$	1.09 × $10^{03}$
**Win**	**0**		**4**		**11**		**13**	

**Table 10:** Use the test results of CEC2013 on 50 dimensions and compare with the original algorithm and other improvements of the original algorithm (bold font indicates optimal data).

Function	BFGO		QBFGO		OBFGO		OQBFGO	
	Mean	Std	Mean	Std	Mean	Std	Mean	Std
F1	1.78 × $10^{04}$	3.37 × $10^{03}$	**−4.45 × $10^{02}$**	**8.79 × $10^{02}$**	1.27 × $10^{04}$	3.11 × $10^{03}$	−1.28 × $10^{03}$	1.35 × $10^{02}$
F2	2.76 × $10^{08}$	7.62 × $10^{07}$	9.89 × $10^{07}$	4.36 × $10^{07}$	1.89 × $10^{08}$	5.50 × $10^{07}$	**8.11 × $10^{07}$**	**2.17 × $10^{07}$**
F3	1.02 × $10^{11}$	3.76 × $10^{10}$	4.95 × $10^{10}$	1.49 × $10^{10}$	7.11 × $10^{10}$	2.37 × $10^{10}$	**4.80 × $10^{10}$**	**1.23 × $10^{10}$**
F4	9.09 × $10^{04}$	1.06 × $10^{04}$	**7.60 × $10^{04}$**	**6.38 × $10^{03}$**	7.97 × $10^{04}$	5.64 × $10^{03}$	8.50 × $10^{04}$	1.40 × $10^{04}$
F5	2.23 × $10^{03}$	1.23 × $10^{03}$	−7.33 × $10^{02}$	1.71 × $10^{02}$	9.09 × $10^{02}$	4.42 × $10^{02}$	**−9.22 × $10^{02}$**	**3.32 × $10^{01}$**
F6	4.85 × $10^{02}$	3.83 × $10^{02}$	−5.87 × $10^{02}$	7.46 × $10^{01}$	2.66 × $10^{01}$	1.91 × $10^{02}$	**−6.61 × $10^{02}$**	**6.82 × $10^{01}$**
F7	−5.94 × $10^{02}$	5.63 × $10^{01}$	**−6.53 × $10^{02}$**	**3.75 × $10^{01}$**	−6.44 × $10^{02}$	3.00 × $10^{01}$	−6.36 × $10^{02}$	3.51 × $10^{01}$
F8	−6.79 × $10^{02}$	4.94 × $10^{-02}$	**−6.79 × $10^{02}$**	**3.47 × $10^{-02}$**	−6.79 × $10^{02}$	4.40 × $10^{-02}$	−6.79 × $10^{02}$	6.37 × $10^{-02}$
F9	−5.33 × $10^{02}$	5.91 × $10^{00}$	−5.34 × $10^{02}$	5.50 × $10^{00}$	−5.35 × $10^{02}$	5.39 × $10^{00}$	**−5.36 × $10^{02}$**	**3.82 × $10^{00}$**
F10	2.48 × $10^{03}$	6.38 × $10^{02}$	5.49 × $10^{01}$	1.92 × $10^{02}$	1.41 × $10^{03}$	3.74 × $10^{02}$	**−1.90 × $10^{02}$**	**1.08 × $10^{02}$**
F11	4.09 × $10^{02}$	8.76 × $10^{01}$	**1.55 × $10^{01}$**	**9.84 × $10^{01}$**	2.62 × $10^{02}$	9.14 × $10^{01}$	4.06 × $10^{01}$	1.25 × $10^{02}$
F12	5.40 × $10^{02}$	1.61 × $10^{02}$	3.33 × $10^{02}$	9.66 × $10^{01}$	3.61 × $10^{02}$	8.92 × $10^{01}$	**2.77 × $10^{02}$**	**1.09 × $10^{02}$**
F13	6.53 × $10^{02}$	1.45 × $10^{02}$	**4.69 × $10^{02}$**	**7.17 × $10^{01}$**	5.42 × $10^{02}$	1.26 × $10^{02}$	4.93 × $10^{02}$	1.03 × $10^{02}$
F14	1.03 × $10^{04}$	7.42 × $10^{02}$	4.63 × $10^{03}$	1.24 × $10^{03}$	1.03 × $10^{04}$	1.13 × $10^{03}$	**4.16 × $10^{03}$**	**8.48 × $10^{02}$**
F15	1.33 × $10^{04}$	1.08 × $10^{03}$	1.23 × $10^{04}$	1.67 × $10^{03}$	1.29 × $10^{04}$	1.04 × $10^{03}$	**1.15 × $10^{04}$**	**1.34 × $10^{03}$**
F16	**2.04 × $10^{02}$**	**5.95 × $10^{-01}$**	2.04 × $10^{02}$	7.13 × $10^{-01}$	2.04 × $10^{02}$	6.16 × $10^{-01}$	2.04 × $10^{02}$	7.81 × $10^{-01}$
F17	1.34 × $10^{03}$	1.27 × $10^{02}$	8.07 × $10^{02}$	1.11 × $10^{02}$	1.18 × $10^{03}$	1.14 × $10^{02}$	**7.52 × $10^{02}$**	**1.18 × $10^{02}$**
F18	1.33 × $10^{03}$	1.13 × $10^{02}$	1.15 × $10^{03}$	1.04 × $10^{02}$	1.22 × $10^{03}$	8.70 × $10^{01}$	**1.13 × $10^{03}$**	**9.88 × $10^{01}$**
F19	1.94 × $10^{04}$	7.63 × $10^{03}$	6.59 × $10^{02}$	1.49 × $10^{02}$	1.08 × $10^{04}$	6.48 × $10^{03}$	**5.47 × $10^{02}$**	**2.58 × $10^{01}$**
F20	**6.24 × $10^{02}$**	**3.69 × $10^{-01}$**	6.24 × $10^{02}$	6.54 × $10^{-01}$	6.24 × $10^{02}$	5.66 × $10^{-01}$	6.24 × $10^{02}$	6.46 × $10^{-01}$
F21	4.22 × $10^{03}$	2.33 × $10^{02}$	2.45 × $10^{03}$	6.23 × $10^{02}$	4.11 × $10^{03}$	2.15 × $10^{02}$	**1.87 × $10^{03}$**	**3.60 × $10^{02}$**
F22	1.27 × $10^{04}$	1.37 × $10^{03}$	7.83 × $10^{03}$	1.52 × $10^{03}$	1.26 × $10^{04}$	1.10 × $10^{03}$	**7.23 × $10^{03}$**	**1.57 × $10^{03}$**
F23	1.62 × $10^{04}$	8.97 × $10^{02}$	1.44 × $10^{04}$	1.41 × $10^{03}$	1.58 × $10^{04}$	7.72 × $10^{02}$	**1.40 × $10^{04}$**	**1.50 × $10^{03}$**
F24	1.39 × $10^{03}$	7.21 × $10^{00}$	1.37 × $10^{03}$	1.55 × $10^{01}$	1.39 × $10^{03}$	1.05 × $10^{01}$	**1.36 × $10^{03}$**	**1.43 × $10^{01}$**
F25	1.49 × $10^{03}$	1.19 × $10^{01}$	**1.47 × $10^{03}$**	**1.05 × $10^{01}$**	1.48 × $10^{03}$	8.20 × $10^{00}$	1.47 × $10^{03}$	1.34 × $10^{01}$
F26	1.68 × $10^{03}$	1.13 × $10^{01}$	**1.63 × $10^{03}$**	**9.01 × $10^{01}$**	1.65 × $10^{03}$	7.93 × $10^{01}$	1.66 × $10^{03}$	1.21 × $10^{01}$
F27	3.46 × $10^{03}$	1.22 × $10^{02}$	3.30 × $10^{03}$	1.42 × $10^{02}$	3.43 × $10^{03}$	8.02 × $10^{01}$	**3.26 × $10^{03}$**	**1.30 × $10^{02}$**
F28	7.14 × $10^{03}$	1.05 × $10^{03}$	**4.74 × $10^{03}$**	**1.76 × $10^{03}$**	5.95 × $10^{03}$	1.05 × $10^{03}$	5.00 × $10^{03}$	2.25 × $10^{03}$
**Win**	**2**		**9**		**0**		**17**	

**Table 11:** Use the test results of CEC2013 on 30 dimensions and compare with mature algorithms (bold font indicates optimal data).

Function	OQBFGO		PSO		GWO		DE	
	Mean	Std	Mean	Std	Mean	Std	Mean	Std
F1	**−1.34 × $10^{03}$**	**7.10 × $10^{01}$**	8.26 × $10^{03}$	3.09 × $10^{03}$	1.43 × $10^{03}$	1.61 × $10^{03}$	7.85 × $10^{04}$	9.86 × $10^{03}$
F2	**4.99 × $10^{07}$**	**1.62 × $10^{07}$**	1.24 × $10^{08}$	7.84 × $10^{07}$	6.62 × $10^{07}$	3.16 × $10^{07}$	6.54 × $10^{08}$	2.07 × $10^{08}$
F3	3.88 × $10^{10}$	1.99 × $10^{10}$	2.61 × $10^{11}$	3.55 × $10^{11}$	**2.14 × $10^{10}$**	**9.31 × $10^{09}$**	1.75 × $10^{13}$	9.25 × $10^{13}$
F4	**4.57 × $10^{04}$**	**6.35 × $10^{03}$**	1.09 × $10^{05}$	4.59 × $10^{04}$	1.01 × $10^{05}$	2.35 × $10^{04}$	2.44 × $10^{05}$	3.50 × $10^{04}$
F5	**−9.36 × $10^{02}$**	**3.33 × $10^{01}$**	4.66 × $10^{03}$	4.50 × $10^{03}$	8.95 × $10^{02}$	1.27 × $10^{03}$	3.03 × $10^{04}$	6.87 × $10^{03}$
F6	**−7.69 × $10^{02}$**	**4.29 × $10^{01}$**	−2.13 × $10^{02}$	3.55 × $10^{02}$	−6.53 × $10^{02}$	1.19 × $10^{02}$	1.20 × $10^{04}$	3.18 × $10^{03}$
F7	−6.33 × $10^{02}$	6.65 × $10^{01}$	−4.16 × $10^{02}$	3.38 × $10^{02}$	**−6.61 × $10^{02}$**	**4.99 × $10^{01}$**	−8.03 × $10^{01}$	6.81 × $10^{02}$
F8	−6.79 × $10^{02}$	6.21 × $10^{-02}$	−6.79 × $10^{02}$	5.10 × $10^{-02}$	**−6.79 × $10^{02}$**	**4.46 × $10^{-02}$**	−6.79 × $10^{02}$	6.59 × $10^{-02}$
F9	−5.66 × $10^{02}$	3.91 × $10^{00}$	−5.64 × $10^{02}$	3.99 × $10^{00}$	**−5.72 × $10^{02}$**	**4.21 × $10^{00}$**	−5.56 × $10^{02}$	1.28 × $10^{00}$
F10	**−3.71 × $10^{02}$**	**5.19 × $10^{01}$**	1.23 × $10^{03}$	7.80 × $10^{02}$	1.80 × $10^{02}$	2.46 × $10^{02}$	7.99 × $10^{03}$	1.37 × $10^{03}$
F11	−1.33 × $10^{02}$	1.01 × $10^{02}$	7.26 × $10^{01}$	1.18 × $10^{02}$	**−2.09 × $10^{02}$**	**3.86 × $10^{01}$**	8.27 × $10^{02}$	1.34 × $10^{02}$
F12	3.83 × $10^{01}$	7.19 × $10^{01}$	1.63 × $10^{02}$	1.24 × $10^{02}$	**−5.04 × $10^{01}$**	**5.97 × $10^{01}$**	9.26 × $10^{02}$	1.40 × $10^{02}$
F13	1.75 × $10^{02}$	7.27 × $10^{01}$	2.84 × $10^{02}$	8.63 × $10^{01}$	**9.79 × $10^{01}$**	**4.08 × $10^{01}$**	1.05 × $10^{03}$	1.74 × $10^{02}$
F14	**2.36 × $10^{03}$**	**6.34 × $10^{02}$**	6.69 × $10^{03}$	7.63 × $10^{02}$	5.49 × $10^{03}$	1.88 × $10^{03}$	7.42 × $10^{03}$	3.63 × $10^{02}$
F15	**5.43 × $10^{03}$**	**7.59 × $10^{02}$**	7.41 × $10^{03}$	8.85 × $10^{02}$	7.33 × $10^{03}$	1.68 × $10^{03}$	8.79 × $10^{03}$	4.15 × $10^{02}$
F16	**2.03 × $10^{02}$**	**6.15 × $10^{-01}$**	2.04 × $10^{02}$	7.14 × $10^{-01}$	2.04 × $10^{02}$	4.93 × $10^{-01}$	2.04 × $10^{02}$	4.53 × $10^{-01}$
F17	**4.89 × $10^{02}$**	**4.85 × $10^{01}$**	9.98 × $10^{02}$	1.56 × $10^{02}$	6.12 × $10^{02}$	5.52 × $10^{01}$	3.21 × $10^{03}$	3.85 × $10^{02}$
F18	**7.66 × $10^{02}$**	**6.62 × $10^{01}$**	1.02 × $10^{03}$	1.52 × $10^{02}$	7.92 × $10^{02}$	5.78 × $10^{01}$	3.34 × $10^{03}$	3.35 × $10^{02}$
F19	**5.26 × $10^{02}$**	**1.29 × $10^{01}$**	3.14 × $10^{04}$	5.24 × $10^{04}$	2.21 × $10^{03}$	2.68 × $10^{03}$	4.05 × $10^{06}$	3.13 × $10^{06}$
F20	6.15 × $10^{02}$	3.54 × $10^{-01}$	6.15 × $10^{02}$	5.61 × $10^{-01}$	6.15 × $10^{02}$	5.82 × $10^{-01}$	**6.15 × $10^{02}$**	**1.17 × $10^{-01}$**
F21	**1.20 × $10^{03}$**	**1.77 × $10^{02}$**	2.51 × $10^{03}$	2.81 × $10^{02}$	2.13 × $10^{03}$	3.89 × $10^{02}$	6.81 × $10^{03}$	5.97 × $10^{02}$
F22	**4.30 × $10^{03}$**	**9.60 × $10^{02}$**	8.29 × $10^{03}$	6.55 × $10^{02}$	7.22 × $10^{03}$	1.74 × $10^{03}$	9.22 × $10^{03}$	3.28 × $10^{02}$
F23	**7.61 × $10^{03}$**	**9.66 × $10^{02}$**	8.77 × $10^{03}$	6.90 × $10^{02}$	7.77 × $10^{03}$	1.57 × $10^{03}$	1.01 × $10^{04}$	3.16 × $10^{02}$
F24	1.29 × $10^{03}$	9.32 × $10^{00}$	1.31 × $10^{03}$	1.17 × $10^{01}$	**1.27 × $10^{03}$**	**1.33 × $10^{01}$**	1.32 × $10^{03}$	5.11 × $10^{00}$
F25	**1.39 × $10^{03}$**	**1.01 × $10^{01}$**	1.42 × $10^{03}$	1.37 × $10^{01}$	1.40 × $10^{03}$	1.19 × $10^{01}$	1.42 × $10^{03}$	4.81 × $10^{00}$
F26	**1.49 × $10^{03}$**	**9.14 × $10^{01}$**	1.58 × $10^{03}$	4.85 × $10^{01}$	1.54 × $10^{03}$	6.37 × $10^{01}$	1.61 × $10^{03}$	1.13 × $10^{01}$
F27	2.51 × $10^{03}$	8.55 × $10^{01}$	2.55 × $10^{03}$	9.05 × $10^{01}$	**2.33 × $10^{03}$**	**1.18 × $10^{02}$**	2.75 × $10^{03}$	3.95 × $10^{01}$
F28	4.24 × $10^{03}$	1.09 × $10^{03}$	5.00 × $10^{03}$	9.93 × $10^{02}$	**3.44 × $10^{03}$**	**6.81 × $10^{02}$**	7.34 × $10^{03}$	5.15 × $10^{02}$
**Win**	**17**		**0**		**10**		**1**	

**Table 12:** Use the test results of CEC2013 on 50 dimensions and compare with mature algorithms (bold font indicates optimal data).

Function	OQBFGO		PSO		GWO		DE	
	Mean	Std	Mean	Std	Mean	Std	Mean	Std
F1	**−1.28 × $10^{03}$**	**1.35 ×$10^{02}$**	3.08 × $10^{04}$	8.75 × $10^{03}$	7.71 × $10^{03}$	3.77 × $10^{03}$	1.78 × $10^{05}$	1.50 × $10^{04}$
F2	**8.11 × $10^{07}$**	**2.17 × $10^{07}$**	3.85 × $10^{08}$	1.63 × $10^{08}$	1.33 × $10^{08}$	4.94 × $10^{07}$	2.60 × $10^{09}$	5.37 × $10^{08}$
F3	4.80 × $10^{10}$	1.23 × $10^{10}$	7.34 × $10^{11}$	1.81 × $10^{12}$	**4.44 × $10^{10}$**	**9.83 × $10^{09}$**	7.11 × $10^{14}$	1.88 × $10^{15}$
F4	**8.50 × $10^{04}$**	**1.40 × $10^{04}$**	1.83 × $10^{05}$	4.50 × $10^{04}$	1.57 × $10^{05}$	2.22 × $10^{04}$	4.06 × $10^{05}$	6.20 × $10^{04}$
F5	**−9.22 × $10^{02}$**	**3.32 × $10^{01}$**	9.15 × $10^{03}$	7.00 × $10^{03}$	1.22 × $10^{03}$	8.02 × $10^{02}$	9.81 × $10^{04}$	1.47 × $10^{04}$
F6	**−6.61 × $10^{02}$**	**6.82 × $10^{01}$**	1.80 × $10^{03}$	1.20 × $10^{03}$	−3.28 × $10^{02}$	1.88 × $10^{02}$	3.32 × $10^{04}$	8.44 × $10^{03}$
F7	−6.36 × $10^{02}$	3.51 × $10^{01}$	6.17 × $10^{02}$	2.65 × $10^{03}$	**−6.64 × $10^{02}$**	**2.27 × $10^{01}$**	1.55 × $10^{04}$	2.24 × $10^{04}$
F8	−6.79 × $10^{02}$	6.37 × $10^{-02}$	−6.79 × $10^{02}$	5.14 × $10^{-02}$	**−6.79 × $10^{02}$**	**2.70 × $10^{-02}$**	−6.79 × $10^{02}$	4.31 × $10^{-02}$
F9	−5.36 × $10^{02}$	3.82 × $10^{00}$	−5.33 × $10^{02}$	4.00 × $10^{00}$	**−5.45 × $10^{02}$**	**5.89 × $10^{00}$**	−5.20 × $10^{02}$	1.74 × $10^{00}$
F10	**−1.90 × $10^{02}$**	**1.08 × $10^{02}$**	4.49 × $10^{03}$	1.63 × $10^{03}$	9.43 × $10^{02}$	4.40 × $10^{02}$	2.02 × $10^{04}$	2.61 × $10^{03}$
F11	4.06 × $10^{01}$	1.25 × $10^{02}$	5.70 × $10^{02}$	9.63 × $10^{01}$	**6.88 × $10^{00}$**	**6.53 × $10^{01}$**	2.43 × $10^{03}$	3.57 × $10^{02}$
F12	2.77 × $10^{02}$	1.09 × $10^{02}$	6.93 × $10^{02}$	1.26 × $10^{02}$	**2.18 × $10^{02}$**	**9.08 × $10^{01}$**	2.32 × $10^{03}$	2.83 × $10^{02}$
F13	4.93 × $10^{02}$	1.03 × $10^{02}$	8.25 × $10^{02}$	1.23 × $10^{02}$	**3.83 × $10^{02}$**	**7.51 × $10^{01}$**	2.40 × $10^{03}$	2.50 × $10^{02}$
F14	**4.16 × $10^{03}$**	**8.48 × $10^{02}$**	1.27 × $10^{04}$	9.64 × $10^{02}$	1.18 × $10^{04}$	3.09 × $10^{03}$	1.37 × $10^{04}$	5.42 × $10^{02}$
F15	**1.15 × $10^{04}$**	**1.34 × $10^{03}$**	1.42 × $10^{04}$	7.84 × $10^{02}$	1.33 × $10^{04}$	2.30 × $10^{03}$	1.60 × $10^{04}$	4.71 × $10^{02}$
F16	**2.04 × $10^{02}$**	**7.81 × $10^{-01}$**	2.05 × $10^{02}$	6.77 × $10^{-01}$	2.05 × $10^{02}$	4.64 × $10^{-01}$	2.05 × $10^{02}$	4.26 × $10^{-01}$
F17	**7.52 × $10^{02}$**	**1.18 × $10^{02}$**	1.81 × $10^{03}$	2.22 × $10^{02}$	9.77 × $10^{02}$	9.22 × $10^{01}$	5.84 × $10^{03}$	3.62 × $10^{02}$
F18	1.13 × $10^{03}$	9.88 × $10^{01}$	1.95 × $10^{03}$	2.84 × $10^{02}$	**1.11 × $10^{03}$**	**6.28 × $10^{01}$**	6.00 × $10^{03}$	4.24 × $10^{02}$
F19	**5.47 × $10^{02}$**	**2.58 × $10^{01}$**	1.82 × $10^{05}$	1.37 × $10^{05}$	2.34 × $10^{04}$	4.37 × $10^{04}$	2.42 × $10^{07}$	8.06 × $10^{06}$
F20	**6.24 × $10^{02}$**	**6.46 × $10^{-01}$**	6.25 × $10^{02}$	3.97 × $10^{-01}$	6.24 × $10^{02}$	5.97 × $10^{-01}$	6.25 × $10^{02}$	4.11 × $10^{-03}$
F21	**1.87 × $10^{03}$**	**3.60 × $10^{02}$**	5.07 × $10^{03}$	5.34 × $10^{02}$	4.07 × $10^{03}$	3.67 × $10^{02}$	1.41 × $10^{04}$	1.54 × $10^{03}$
F22	**7.23 × $10^{03}$**	**1.57 × $10^{03}$**	1.55 × $10^{04}$	9.99 × $10^{02}$	1.43 × $10^{04}$	2.97 × $10^{03}$	1.56 × $10^{04}$	4.47 × $10^{02}$
F23	**1.40 × $10^{04}$**	**1.50 × $10^{03}$**	1.61 × $10^{04}$	8.43 × $10^{02}$	1.45 × $10^{04}$	1.97 × $10^{03}$	1.75 × $10^{04}$	5.63 × $10^{02}$
F24	1.36 × $10^{03}$	1.43 × $10^{01}$	1.40 × $10^{03}$	1.44 × $10^{01}$	**1.35 × $10^{03}$**	**1.08 × $10^{01}$**	1.42 × $10^{03}$	8.30 × $10^{00}$
F25	**1.47 × $10^{03}$**	**1.34 × $10^{01}$**	1.52 × $10^{03}$	1.46 × $10^{01}$	1.51 × $10^{03}$	1.64 × $10^{01}$	1.52 × $10^{03}$	7.46 × $10^{00}$
F26	1.66 × $10^{03}$	1.21 × $10^{01}$	1.65 × $10^{03}$	7.61 × $10^{01}$	**1.63 × $10^{03}$**	**4.21 × $10^{01}$**	1.70 × $10^{03}$	5.78 × $10^{00}$
F27	3.26 × $10^{03}$	1.30 × $10^{02}$	3.43 × $10^{03}$	1.23 × $10^{02}$	**3.06 × $10^{03}$**	**1.55 × $10^{02}$**	3.71 × $10^{03}$	5.72 × $10^{01}$
F28	5.00 × $10^{03}$	2.25 × $10^{03}$	8.98 × $10^{03}$	1.15 × $10^{03}$	**4.93 × $10^{03}$**	**1.18 × $10^{03}$**	1.73 × $10^{04}$	1.33 × $10^{03}$
**Win**	**16**		**0**		**12**		**0**	

**Table 13:** Comparison results of six algorithms for solving CVRP.

	BFGO	QUATRE	PSO	GWO	DE	OQBFGO
A-n32-k5	1018.1	860.98	968.38	1266.4	1203.9	**788.51**
A-n33-k5	701.59	1051.2	878.13	912.79	981.08	**676.42**
A-n33-k6	970.72	939.29	934.09	969.7	1049.4	**805.36**
A-n34-k5	1006	874.82	1050.3	849.88	1204.8	**831.19**
A-n36-k5	962.01	792.99	973.11	780.72	1058.2	**723.87**
A-n44-k7	1311.9	1610.3	1262	1683.4	1596.2	**1123.2**
A-n45-k6	1859.6	1842.9	1729.8	1944.7	1949	**1296.9**
A-n53-k7	2026	1885.3	1411.2	2055.1	2145.9	**1366.8**
A-n60-k9	2513	2358.2	2439	2738.6	2517.6	**1770.4**
A-n63-k10	2266.2	2642	2667.5	2390.4	2860.7	**1754.5**

## Data Availability

Not applicable.
